# Security and Trust Management in the Internet of Vehicles (IoV): Challenges and Machine Learning Solutions

**DOI:** 10.3390/s24020368

**Published:** 2024-01-08

**Authors:** Easa Alalwany, Imad Mahgoub

**Affiliations:** 1 College of Computer Science and Engineering, Taibah University, Yanbu 46421, Saudi Arabia; ealalwany2020@fau.edu; 2 Electrical Engineering & Computer Science, Florida Atlantic University, 777 Glades Road, Boca Raton, FL 33431, USA

**Keywords:** Internet of Vehicles, Internet of Things, machine learning, security, trust

## Abstract

The Internet of Vehicles (IoV) is a technology that is connected to the public internet and is a subnetwork of the Internet of Things (IoT) in which vehicles with sensors are connected to a mobile and wireless network. Numerous vehicles, users, things, and networks allow nodes to communicate information with their surroundings via various communication channels. IoV aims to enhance the comfort of driving, improve energy management, secure data transmission, and prevent road accidents. Despite IoV’s advantages, it comes with its own set of challenges, particularly in the highly important aspects of security and trust. Trust management is one of the potential security mechanisms aimed at increasing reliability in IoV environments. Protecting IoV environments from diverse attacks poses significant challenges, prompting researchers to explore various technologies for security solutions and trust evaluation methods. Traditional approaches have been employed, but innovative solutions are imperative. Amid these challenges, machine learning (ML) has emerged as a potent solution, leveraging its remarkable advancements to effectively address IoV’s security and trust concerns. ML can potentially be utilized as a powerful technology to address security and trust issues in IoV environments. In this survey, we delve into an overview of IoV and trust management, discussing security requirements, challenges, and attacks. Additionally, we introduce a classification scheme for ML techniques and survey ML-based security and trust management schemes. This research provides an overview for understanding IoV and the potential of ML in improving its security framework. Additionally, it provides insights into the future of trust and security enhancement.

## 1. Introduction

Today, with recent innovations in technology, the ability of different devices we use in our daily lives to connect to the internet, communicate, and exchange messages has rapidly increased. A large number of devices of different categories currently extend the internet to almost every part of the world. These devices provide different services via communication with other devices. The devices, whether a smartphone, vehicle, or vending machine, are capable of connecting to the internet and sharing data. The Internet of Things (IoT) provides various services to users in the whole network system. The IoT is also expected to increase automation levels. The IoT increases the efficiency of smart health, smart cities, and smart transportation when it is integrated with these services. The objective of this smart environment is to save money, time, lives, and energy [1,2,3].

The IoT has been implemented in the transportation industry by specialists through the development of wireless and mobile communication technologies. As a result, wireless networks have been steadily deployed on vehicles and roadways, allowing vehicles to share information with each other and with infrastructure. To make these vehicles more intelligent and secure, they are being equipped with external and internal sensors. External sensors are attached outside of the vehicle and include cameras and parking sensors, while internal sensors include automotive sensors such as brake sensors, fuel sensors, and tire pressure sensors. Vehicles equipped with sensors that communicate through a mobile and wireless network are considered part of the IoT and are referred to as the Internet of Vehicles (IoV). IoV integrates two technologies, vehicular ad hoc networks (VANETs) and the IoT, to take steps toward intelligent transportation [4,5,6,7,8]. The IoT mobilizes IoV to produce a revolution in the field. IoV is an open, integrated network system with significant controllability, manageability, and credibility that has evolved from the IoT. Numerous vehicles, users, things, and networks allow nodes to communicate information with their surroundings via various communication channels. IoV is considered one of the most significant IoT applications in the area of automobiles [9,10,11,12,13,14]. Driving safety is the main goal of many applications of IoV environments. IoV has its own set of challenges, particularly in terms of security and trust, since it has to provide highly important security functions. As with other technologies, IoV has many security vulnerabilities. Hackers could control vehicles if vulnerabilities in IoV are exposed, which could lead to traffic accidents. The security of IoVs is a critical issue for the safety of drivers and anyone using the roadways. The need for data security will increase as the number of vehicles connected to the public Internet increases.

A trusted environment is a method of achieving security in IoV networks; hence, trust is an essential component of security. Messages exchanged between vehicles and their surroundings must be legitimate since trust must be established between vehicles. The detection and revocation of malicious vehicles, as well as their communications, is critical to providing a safe IoV environment. Before accepting and sending a message received by a legitimate vehicle, the vehicle should examine its trustworthiness and legitimacy. Some aspects of the V2X paradigm have been known to prompt major security concerns as well as lead to road accidents and traffic congestion. Users of vehicular networks aim to avoid any infringement on their privacy and any disclosure of private information. It is critical that users can also ensure that the data they receive are accurate. Traditional methods may not be able to address all these concerns or safeguard current IoV scenarios. It is important for security requirements such as integrity, confidentiality, and availability to be carefully considered by organizations. During an interaction between nodes, we define trust management as a collection of steps whereby a node attempts to establish trust with another node. Trust factor is a property measured as a quantifiable belief by a trustor node and trustee node and ensures that the negative impacts of malicious and selfish nodes are significantly minimized. The categories of trust properties are subjective and objective trust, local and global trust, context-based trust and history-based trust as well as direct and indirect trust. For the safety of their users, IoV systems must be protected from any form of cyberattacks that may interfere with any operation. To boost IoV security, certain steps are undertaken, the main one being trust management; therefore, many research works have focused on integrating trust management in the vehicular environment. Nowadays, the most promising technology in the wireless network field is machine learning (ML), and ML techniques are being used in a wide range of applications in wireless networks [15,16]. Recently, many solutions based on ML technologies in security for wireless networks have been proposed [17].

IoV networks aim to improve traffic safety and efficiency. A secure and trusted environment is essential for vehicles to be able to exchange data with each other. Modeling trust and security in IoV networks has been a challenge because these networks are dynamic, depend on sensitive communication, and are naturally open. In IoV environments, dishonest vehicles and attacks endanger the lives of drivers, passengers, and pedestrians. Security solutions and trust management have been employed to make sure that vehicular networks are safe and reliable. The use of ML techniques in IoV environments is rapidly increasing, yet the security implications of their integration with IoV have received little attention. ML techniques are gaining increased attention in trust and security research, and it would be interesting to investigate the relevant aspects of this research to create a secure and trusting IoV environment. ML techniques, such as supervised learning, unsupervised learning, and reinforcement learning, are reviewed in this survey to enhance security and trust management in vehicular networks. Our contributions are:

- We provide a detailed description of the concept of IoV that provides an overview and covers the architectures and types of connections in IoV.
- We explain the fundamentals of trust evaluation and its features in IoV.
- Security is a significant factor in an IoV environment, and serious security concerns of IoV are discussed in this survey.
- An IoV environment requires various security requirements to ensure constant safety and security.
- The survey discusses and identifies various security attacks including attacks on authentication, confidentiality, availability, integrity, secrecy, and routing.
- We present possible trust and security solutions for IoV environments by mainly focusing on classification using three types of ML models (supervised learning, unsupervised learning, and reinforcement learning).

This paper is organized into the following sections. Section 2 provides background information. Section 3 discusses related surveys. Section 4 describes the concept of IoV. Section 5 examines solutions based on ML and traditional approaches for security and trust in IoV. Section 6 concludes the paper.

## 2. Search Methodology

A structured methodology was utilized to conduct a comprehensive review of the literature on the Internet of Vehicles (IoV), with a focus on security, trust, and the potential role of machine learning (ML). The method started with a search for keywords that accurately describe the key concepts of the study, such as “Internet of Vehicles”, “IoV security”, “IoV trust”, “machine learning”, “ML-based security”, “ML-based trust”, and “IoV challenges”. Google Scholar, IEEE Xplore, Springer, MDPI, and the ACM Digital Library were the academic search engines used to ensure that our researchers had access to the most recent and significant literature in their respective fields. In order to ensure the timeliness of the review, a certain publishing date range was delineated, covering 2017 to 2023. This range was chosen due to the notable advancements observed in the fields of IoV and machine learning throughout this period. The selection criteria for this survey were designed to ensure the survey’s concentration and applicability. This study only incorporates research specifically focused on ML-based approaches for addressing security and trust concerns within an IoV environment. Any study that was outdated and no longer relevant to the current state of security and trust in IoV utilizing ML was excluded from the survey. This ensured that the survey accurately represented the most recent advancements in this field of study. Furthermore, in order to ensure the precision and effectiveness of the survey, works that lack substantial relevance or significance for developing the current landscape of ML in IoV were also excluded. After implementing these exclusion criteria, 50 publications related to the classification of security and trust solutions for IoV were selected for this review.

## 3. Background

### 3.1. Trust Management in IoV

Trust is described as the probability that an individual will anticipate the behavior of another peer based on the behavior of the peer and the individual’s well-being. This definition comes from the field of psychology [18]. It is worth noting that trust is significantly influenced by the subject’s viewpoint. As a result, trust indicates the trustor’s confidence that the trustee will act in his or her best interests [19]. According to sociological research, reciprocity and cooperation among persons in social interactions are believed to build trust among individuals [20]. In the computer science field, trust is defined as a trustor’s conviction in the reliability of the target node, with the goal of achieving a trust objective under particular conditions [21]. Trust can also be viewed as an assessment made by an evaluator, which is influenced by past experiences with a particular entity and the perspectives of other trustworthy sources [22]. Trust management strategies are commonly employed to secure many network environments. To comprehend trust management approaches, it is also important to understand the basic definition of the trust concept. There is a distinction between trust and trustworthiness, which is a trait that describes reliability. Trust and reputation can be used interchangeably and at times can cause confusion. The reputation of a specific node in the network environment is defined as the opinion of that node that has been built based on recommendations of other network nodes that are either direct or indirect neighbors of the node in question. Trust concepts demonstrate the relationship existing between a trustor and trustee and that the trustor believes the trustee will fulfill its obligations. The success of trust relationships result in security and optimistic feelings, while their failure result in mistrust and insecurities. In the computer science community, the trust concept has been proven to be ideal for the protection of networks [23,24,25].

#### 3.1.1. Trust Properties

Consideration of the concept that trust is a relationship between two entities, the trustor and trustee in this case, leads to the emergence of these attributes. In this instance, the trustee becomes the trustworthy entity. The trust properties are shown in Table 1.

#### 3.1.2. Components of Trust

Direct trust: Because of the interactions between the trustor and the target vehicle, this component displays a trustor’s direct observations of a target vehicle [26]. Knowledge, in the context of some studies, can mean any information the trustor learns about the trustee [27]. This normally makes use of parameters that are dependent on the nodes and services involved. Although direct trust is more important than indirect trust, when evaluating a vehicle, a combination of the two should be considered [28]. The indirect trust component is used to display the opinions of the trustor’s neighbors about the trustee, who is the target node. Indirect trust usually considers the node’s previous encounters and experiences. In some circumstances, researchers employ both experience and reputation to explain the indirect observation. All previous experiences with a target node are included in reputation, which contributes to the formation of a global opinion about the node under observation. Experience, on the other hand, is frequently based on the interaction between the trustor and the trustee, and it strongly relies on the trustor’s confidence in the trustee’s competence to complete a task [21].

#### 3.1.3. Attributes of Trust

Various trust attributes have to be considered when computing the components of trust mentioned earlier.

- Similarity: This refers to the degree to which two vehicles are similar in terms of content and services. Euclidean distance is often used to describe the similarity of messages or vehicles in the literature. The direction of travel of the two nodes, which is usually the location based on trajectory similarity, is known as Euclidean distance [29].
- Timeliness: The attribute of timeliness relates to how recently the two vehicles have interacted with each other. It is usually determined by adding the current time to the time when the interaction happened. Maintaining the timeliness of data and the trust score contributes to higher levels of trust; however, old data reveal an outmoded trust value, which can lead to negative consequences [30].
- Duration of Interaction: This refers to the length of interaction among the two nodes. Longer interactions allow the entity to learn more about the other entity’s conduct and capabilities, and as a result, long interactions have been seen to lead to better interactions among entities, which leads to higher trust levels [31].
- Familiarity: This attribute exhibits the level of acquaintance the two vehicles have with one another. This feature was derived from social networks, and it is worth noting that increased familiarity leads to increased trust in interpersonal relationships. Higher familiarity with the trustee is frequently a reflection of the evaluator’s past understanding and knowledge of the trustee [32].
- Packet Delivery Ratio: This can be described as the degree of connection between the trustor and the trustee. The only criterion required to calculate direct trust toward a trustee is the packet delivery ratio. In the literature, this is typically referred to as the packet transmission rate between nodes. Furthermore, it is a main goal in the development of trust models and a key criterion for detecting harmful activity [33].
- Frequency of Interactions: On a regular basis, the trustor and trustee communicate with one another., and this is measured by the frequency of their interactions. When two nodes communicate, they can learn each other’s communication and behavioral patterns to improve trust computations [31].

#### 3.1.4. Trust Metrics

The proposed trust management approaches have been known to apply different metric methods to measure and evaluate trust value. These methods are:

- Reputation-based metrics: This type of trust metric calculates trust value from the recommendations given by specific nodes in the network. These network nodes may have similar opinions about the node that has been propagated within the network. This metric method considers major opinions or global feedback regarding the node.
- Knowledge-based metrics: This technique calculates trust value based on direct or previous experience that the node has or has gained from another specific node. These metrics help identify selfish nodes that may be part of a network.
- Expectation-based metrics: These metric methods involve a node determining the trust of another node based on how it expects the node to act. Its expectations are based upon previous interactions with the node, received suggestions, or the initial prediction in the case of no prior communication.
- Node-properties-based metrics: Trust calculations make use of the main parameters of proximity such as location and distance with the considered node.
- Environmental-factors-based metrics: When measuring an IoV network, this metric takes into account environmental factors, including network density and topology.

#### 3.1.5. Trust Computation

When trust has been established, it has to be managed throughout the duration of the target node interaction. Some commonly considered modules in trust computation are:

- Trust Propagation: This module assists in establishing the trustworthiness of various communication system nodes based on previously established worthiness values while collaborating. It combines features from both a centralized approach, where trust is granted to entities through a single, trusted node or mechanism, and a decentralized system, where no one entity acts as a central point of control. The module’s main features are trust transitivity and trust fusion. Instead of determining each individual entity’s trust, resource computation costs in this module can be decreased by measuring trust value in a propagating network [34].
- Trust Aggregation: Multiple network pathways can be used to disseminate different versions of a node’s trust value. When it receives diverse trust values for the node, this module aims to define a singular value based on the sum of data received. The Bayesian model, weighted sum approaches, and fuzzy logic are the most commonly used aggregation strategies. The primary principle for composing trust from the path of trust for various received values is trust aggregation.
- Trust Update: This refers to the process of bringing trust values up to date, and it can be divided into three schemas:
  - Event-driven Trust: this is where node trust is adjusted after an event or during the occurrence of a transaction.
  - Time-driven trust: this is where the aggregation scheme is used to adjust the node trust value within a determined time period.
  - Continuous trust update: this is mostly used to protect integrity and is used to regulate one single node task.
- Trust Prediction: the purpose of this module is to predict trust connections between entities by utilizing selected criteria. This module predicts whether trust can develop between trusted nodes.
- Trust Evaluation: This module often contains sections on experience, suggestions, and global knowledge. Requesting node neighbors provides experience, which is continually updated in the table of trust, from which it is communicated as a recommendation trust node. The assessed trust value is linked to the global knowledge component.
- Trust Formation: This is the module where the trust formula is defined. To define how trust can be computed, it is necessary to establish the set of trust qualities and metrics considered by the trust formula. For the formation of the trust formula, the two trust categories of multitrust and single trust must be defined.

#### 3.1.6. Trust Management Approaches

Entity-based approaches, data-based approaches, and integrated approaches are the three types of trust management approaches. The trust concept is related with network nodes in an entity-based approach. These methods are used to assess how trustworthy the nodes in a network are that send and receive data. Malicious nodes can subsequently be excluded from the network or isolated based on the evaluation of their trust ratings. Social trust interactions are inherited by certain existing entity-based techniques in the literature. These methods primarily consider measures that are based on a person’s reputation. As a result, the trust formula used to generate the trustee node’s trust value is mostly dependent on previous knowledge-related metrics as well as traded recommendations among various entities. Other studies look at comprehensive trust, considering not only reputation-based indicators but also the similarity aspect. In data-based systems, the concept of trust is closely associated with the integrity of the generated message, hence necessitating the assurance of data authenticity in these solutions. Utility refers to a specific helpful act or the worth of the created event compared to other actions in the same context, and it is an essential factor in determining data content worthiness. The proximity time, the type of incident that occurred, and the role of the vehicular node are some of the major trust elements considered in data utility assessments. Information-oriented methods and event-oriented methods are two types of data-driven approaches. Similarity is a term that describes how the contents of shared data are similar in terms of time and proximity criteria, and it is used to determine the value of data trust. Similarity aids in reducing the amount of data disseminated, ensuring that only meaningful information is broadcast. The primary principle of the combined trust management technique relies on entity and data-sharing trustworthiness. This module is more efficient when it comes to trust computation. The assessment of data trust value is aided by entity trustworthiness.

An example was provided to address the issue of injecting inaccurate information into safety-related events in VANETs, in which a similarity-based solution was proposed [35]. The calculation of a similarity rating is based on periodic beacons that contain position and speed information. An Echo procedure was also used to acquire a trust rating and to validate the reports by observing the vehicle’s normal and expected behavior in relation to the reported occurrence. In a VANET presented in [36], a multidimensional trust system was created for the agents. In order to require further feedback from trustworthy nodes, the system’s trust values must be maintained. When determining trustworthiness, authors take into account variables including experience, majority opinion, and priority criteria. These schemes classify nodes as having an authority role, an expert role, a seniority role, or an ordinary role. It is also worth noting that the experience factor is calculated as a result of the number of encounters. To deal with behavioral changes, the forgetting factor is introduced. Each node communicates its trustworthiness to other entities through trust messages sent to an authentic infrastructure, which later becomes a component of the reputation management center, where node trust is collected [37]. Authentic infrastructure filters trust communications based on statistical regularity, and each node then has access to updated trust data from the reputation center. Historical trust can be determined with the use of authentic center recommendations [38]. Platoon head vehicles are ranked using the reputation criteria. The system model includes servers that are used to assess the trust of vehicles, and reputation values are calculated using feedback from user vehicles. The use of iterative filtering eliminates any malicious user vehicle feedback. The server node then suggests a safe platoon leader vehicle.

### 3.2. Machine Learning

ML is a subfield of artificial intelligence that uses data and previous decisions to predict the future with high accuracy. ML comes in three forms: supervised, unsupervised, and reinforcement learning [39]. Highly accurate results can be ensured through the use of ML for trust evaluation in instances in which big data are processed. The high accuracy in trust evaluation when one uses big data is due to the adequate data sources big data provide [40]. Inaccurate evaluation results are common in instances in which scientists decide to use traditional trust evaluation methods in social networks as well as many other large-scale networking instances, as such instances have sophisticated data structures and generate and process vast volumes of data, thereby introducing enormous complexities. However, when it comes to handling and processing big data, using ML as the primary method provides enormous specific advantages. For trust evaluation, when dealing with big data, ML takes the lead compared with other methods in terms of the appropriateness dimension [41]. From an artificial intelligence perspective, massive convenience is evident in trust evaluation when ML is used. Artificial intelligence uses ML as its primary technology, with the simulation of human behaviors using computers being an example of a perfectly accomplished task using ML. A model for successor computations is obtained through the use of data or experiences that are already available. There is an almost perfect fit between this process and human thinking patterns. Since human beings’ subjective behavior items include trust patterns, the use of ML for trust evaluations is appropriate. ML artificial intelligence models and human behavior simulations using computers provide an almost perfect process for emulating human thinking patterns. The ML-based trust evaluation process is concise and instructive. There is usually a high degree of certainty in using ML-based trust evaluation to solve basic processes. Data processing, model selection, and final model determination are the three rough division steps of ML’s general process. The data processing step involves transforming available raw data into useful features or turning dirty raw data into meaningful features. It involves the use of, among many other methods, data cleansing, feature selection, and data fusion; these are methods that appropriately minimize high dimensions, missing values, noise, and values that are repeated. In the model selection step, the most appropriate trust evaluation model-building algorithm is selected from the many available ML algorithms. The final model selection step involves adjusting parameters and selecting the best performance achievement enabling parameters for the selected algorithm. The performance of the selected algorithm is usually significantly affected by the availed parameters’ configurations, as the algorithm has a set of parameters that should be set for its run to deliver expected results for the given input. Existing experience-based decision-making or human decision-making processes are what the ML-based trust evaluation process simulates. In addition to this process being easily understandable, implementing it is highly effective and easy. ML has a wide range of applications, including fraud detection in finance [42], personalized learning in education [43], disease diagnosis in healthcare [44], and climate modeling in environmental research [45]. ML helps solve challenges related to IoV, especially traffic flow prediction and optimization. The authors in [46] highlighted the importance of timely and reliable traffic flow information for ITS deployment. Traffic flow predictions are accurate using ML algorithms and historical and real-time traffic data. This predictive capability, which is difficult to acquire using traditional approaches, is useful for a variety of IoV applications, such as traffic congestion mitigation, fuel consumption reduction, and location-based services. In addition, the authors in [47] demonstrated how machine learning techniques can improve routing strategies in vehicular networks, including implementing a software-defined networking (SDN) architecture that incorporates a neural network (NN)-based mobility prediction. This approach guarantees uninterrupted connectivity and reduces latency [48], as well as using Q-learning-based hierarchical routing instead of traditional routing tables. This uses self-constructed adaptive Q-value tables that are based on local traffic flow. This approach enables them to achieve high delivery rates and balance network load [49]. These examples demonstrate how machine learning can effectively handle the many forms of communication and Quality of Service (QoS) needs in automotive networks, beyond the performance limitations of conventional networking systems. Moreover, the authors in [50] demonstrated the effectiveness of machine learning in predicting traffic flows, a typical time-series challenge. Because traffic flows are stochastic and nonlinear, conventional methods that rely on autoregressive moving averages frequently fail to account for these characteristics. Effectively enhancing the accuracy of predictions has been accomplished with the help of ML techniques such as kNN and support vector regression (SVR). A fundamental endeavor in intelligent transportation systems, behavior prediction is another area in which ML performs well. The authors in [51] showed that SVMs can predict lane changes. Overall, machine learning in the environment of IoV has shown its adaptability and efficacy in solving a wide range of challenges associated with predicting traffic patterns, optimizing routes, and forecasting behavior in vehicular networks.

#### 3.2.1. Supervised Learning

Supervised learning (SL) can be categorized into regression and classification. In the classification model, the output is categorical. The most common classification models include neural networks, Naïve Bayes, support vector machines, decision tree, and K-nearest neighbor. As for the regression model, the output is continuous. Logic regression methods are commonly used in secure vehicular networks [46,52,53].

#### 3.2.2. Unsupervised Learning

Unsupervised learning (UL) is the opposite of supervised learning because there are no used labels for the dataset. Unsupervised learning can be categorized into clustering and dimensionality reduction applications The most common clustering models are k-means and the Hidden Markov Model, which are commonly used to secure vehicular networks [52,54].

#### 3.2.3. Reinforcement Learning

Reinforcement learning (RL) algorithms fall into two categories: model-free RL algorithms and model-based RL algorithms. Model-free RL algorithms use policy optimization and value-based algorithms as the main approaches. The primary objective of these algorithms is to come up with a strategy that helps individuals gain the best results in the long term. In value-based algorithms, the temporal difference stands for the place where the agent studies the environment and learns the most accurate method for predicting a variable’s value over a certain period of time. The learned state values are then used to improve the state of the environment. The temporal difference performs refreshes depending on the information obtained from the current assessment. In Q-learning, the Q stands for quality. The agent uses a Q-table to choose the best action for each state. It also helps with increasing the rewards generated from all the ideal actions. The State–Action–Reward–State–Action (SARSA) algorithm learns Q values depending on the functions performed by the current system. Most importantly, the Deep Q-Network (DQN) algorithm creates a matrix that enables the working agent to locate the best action so as to maximize future rewards. It should be noted, however, that an increase in the number of states and actions leads to a more complex and time-consuming Q-table characterization [55]. The primary objective of RL is to identify the circumstances under which the output of the IoT system is most highly rewarded [56]. Below are some of the terms used in this process: Agent: the elements that perform the activities. Environment: the circumstances surrounding the agent. State: the present circumstances. Reward: the response of the environment. Value: the long-term reward.

### 3.3. Security Requirements

Security has become a critical issue in the IoT due to the large amount of data being transmitted, the different technologies used, and the development of cloud computing systems. IoT systems are particularly vulnerable when data are transmitted from a user interface to a cloud-based service, as attackers can use loopholes in transmission links to manipulate the data. Moreover, they can take charge of cameras, the brake system as well as the alert system. In such a case, the people inside the vehicle have no control over what the vehicle can do. This is what makes IoV a high-risk technology. Despite efforts to address the challenges presented by IoV, new and more sophisticated challenges keep popping up. The various security threats are a result of the frequent availability of large amounts of data in IoV. Among the many devices and nodes involved in IoV are data, access points, base stations, sink nodes, and backbone points, making data collection security crucial. To guarantee data security, IoV experts must ensure the data are authenticated and made confidential. Other requirements include integrity, authorization, and nonrepudiation [57].

**Figure 1:**
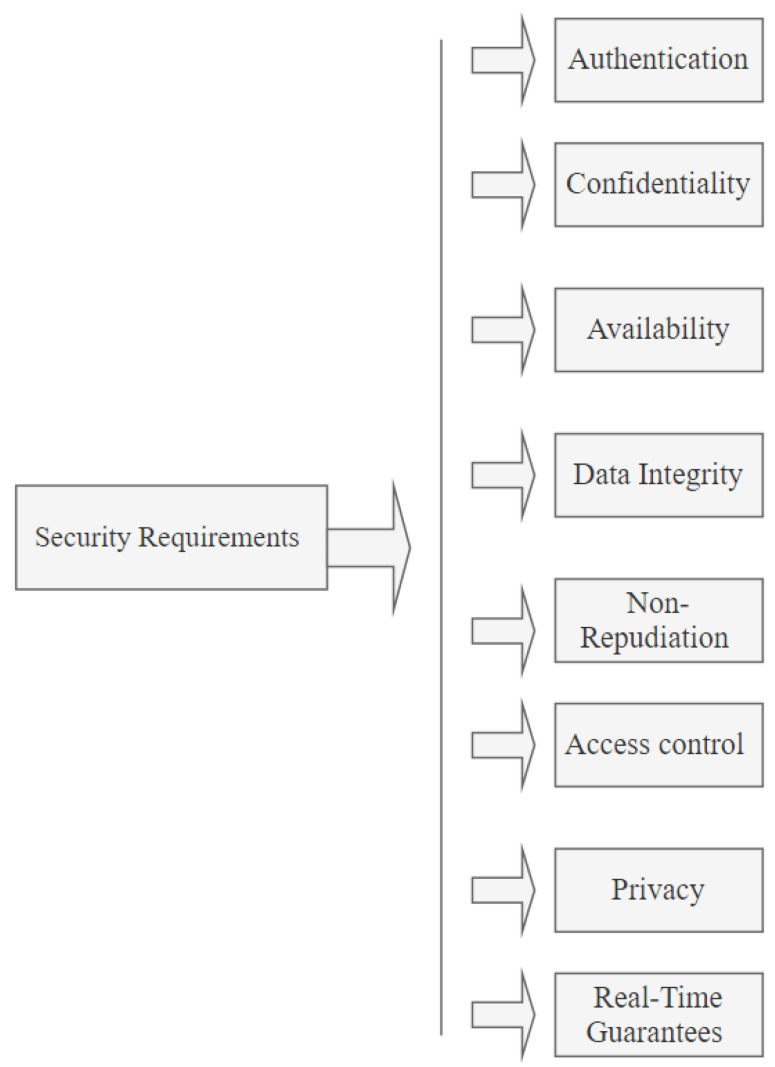
Security requirements in IoV.

Security requirements are measures for identifying how secure an IoV network is. While IoV is vulnerable to multiple threats, researchers have identified some of the most vulnerable areas and how they can be protected from network attacks [57,58,59,60]. Below are some of the security aspects that must be considered. Figure 1 illustrates the security requirements including authentication, confidentiality, availability, data integrity, nonrepudiation, access control, privacy, and real-time guarantees.

#### 3.3.1. Authentication

The system should not allow any form of imitation of a vehicle or vehicles trying to send information. The sensor that sends the data must be the vehicle it claims to be. The receiving sensor should also be able to differentiate between the true sender and a fake sender by scanning the ID of the sender. The chances of a masquerading node acting in the same way as a legitimate node are extremely high. As such, communication between nodes must be authenticated. Every sender must have a unique ID that can be verified through keys or passwords [61].

#### 3.3.2. Confidentiality

Sensitive information passes through the communication channels in IoV networks. This information can have serious effects if it gets into the wrong hands. As such, an efficient system should ensure that sensitive information is secure and only the intended users can access it. Confidential information should be protected at all costs. Encryption has proven to be an effective solution in this endeavor.

#### 3.3.3. Availability

The number of vehicle owners has increased in the past ten years. As such, there is a high likelihood of a high number of IoV users within a particular region at the same time. For this reason, network breakdowns cannot be ruled out, especially during peak hours when everyone is rushing to go home or to work. Hence, an effective system will be available at all times to all legitimate users. The entire IoT system is based on information dissemination. For this reason, information should be made available when needed; otherwise, if there are delays, it might be of no help. It is for this reason that group signature was developed to solve the availability issue.

#### 3.3.4. Data Integrity

The information being delivered from one node to another should not be altered in any way. The content received should match what was sent. In communication that goes through various channels, malicious nodes can tamper with the message or send the wrong signals. Such tampering is dangerous because it might result in messages being interpreted differently. To ensure that the network’s integrity is intact, digital signatures can be used. The content of the message being passed from one node to the other may save a life or cause damage if modified [62].

#### 3.3.5. Nonrepudiation

Nonrepudiation not only detects compromised nodes but also prevents the sender or receiver from denying the transmitted message. Coordination and cooperation among users of an IoT network within a particular range are crucial. For instance, information on an emergency or an accident should be communicated promptly to identify the person responsible. As such, if a user denies a sent message, the user can jeopardize the lives of other users.

#### 3.3.6. Access Control

Access control is comparable to the police in that it ensures that every participating node performs its functions according to its roles and privileges. An efficient system must have an access control panel.

#### 3.3.7. Privacy

A driver’s daily routine is one of the types of private information that should not be made public. No unauthorized access to the network should be allowed since it may put people’s lives at risk [63].

#### 3.3.8. Real-Time Guarantees

Applications used in IoV are designed in such a way that they are time sensitive. For this reason, they have to disseminate information when needed and at the right time. The failure to deliver information in a timely manner can lead to accidents and unnecessary delays.

## 4. Related Surveys

The authors in [64] reported different trust management schemes based on three types of models: entity-centric trust models, data-centric trust models, and combined trust models: a multifaceted trust, a trust and reputation infrastructure-based proposal, a distributed trust, a deterministic approach, a trust model based on various factors of a message, a voting system based on distance from the event, categorized decentralized trust management, an evaluation scheme, and an attack-resistant trust management scheme. They did not, however, present an ML scheme to enhance trust. In the survey in [65], security aspects of IoV, including security requirements and challenges, were considered. Various security threats and existing security solutions for each threat were explored. However, neither ML-based solutions nor trust management were examined in this analysis.

ML-based trust evaluations are free from the inadequacies of traditional trust evaluation methods, as they can carry out trust evaluation to establish a trust model using data about other available trust-related features. ML-based trust evaluations are capable of finding substitute data for the unavailable indirect recommendation and direct historical interaction information in newcomer trustees. ML methods aim to improve the trust evaluation’s accuracy compared with traditional trust evaluation methods [66]. Based on IoV’s security requirements, such as authentication and availability, the authors of [67] classified various security attacks and suggested possible solutions. ML-based solutions were not demonstrated.

Many different trust management models were examined in the survey in [68], and the authors specifically focused on IoVs. One of the intelligent solutions suggested for trust management is the use of context awareness. The authors attempted to demonstrate the potential benefits of context awareness in vehicular networks; however, the authors did not consider ML as a potential solution to the problems of trust and security in IoV. The observation of genetic algorithms (GA), one type of optimization technique, is the focus of reference [69], which aimed to improve the security of IoV networks through the observation of GA. According to the survey, GA is frequently utilized to improve the security of IoV comparisons via various swarm intelligence optimizations. The utilization of the GA in an IoV security system was carried out to improve the accuracy of the network as well as the detection of malicious nodes. However, this survey omitted the use of ML and the improvement in IoV trust.

In [70], the authors discussed the use of ML solutions in VANET such as applications, routing, security, resource allocation and access technologies, mobility management, and integrated architectures. The challenge of trust in the VANET environment was not discussed. In [50], the authors focused on the proposed ML method for ITS challenges. Security and trust were not addressed, but they addressed ML-based works from the point of view of vision-based perception, infrastructure, and resource management as well as the prediction of traffic flow, the behavior of vehicles and users, and the road occupancy of ITS. In [71], the authors investigated the potential integration of Digital Twins (DTs) into IoV, focusing on improving the system design without mentioning machine learning or trust. In [72], the security challenges within vehicular networks are discussed primarily, with an emphasis on attacks and preventive measures, but without explicit mentions of machine learning or trust. In [73], the author examined the security challenges of IoV environments, emphasizing various attack types and the need for privacy preservation and strong authentication, thereby contributing to discussions on security but without ML techniques.

**Figure 2:**
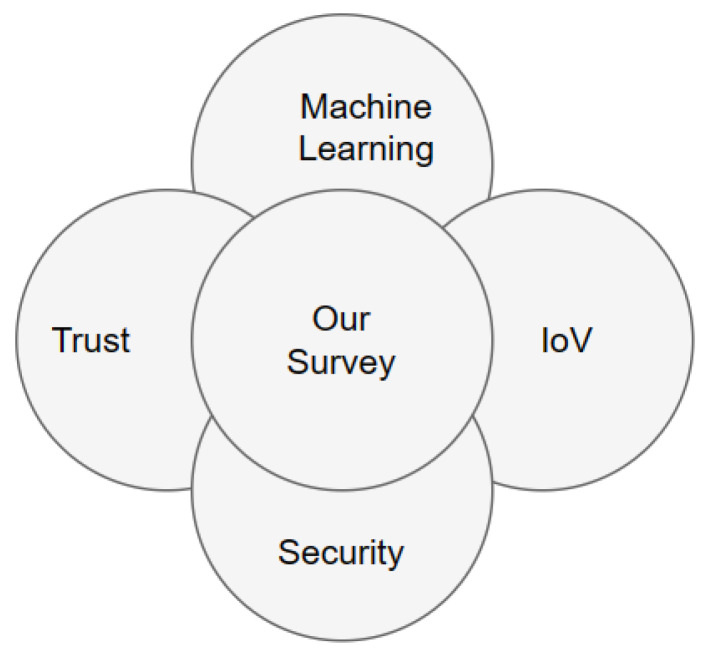
The scope of our survey.

We provide a comprehensive discussion of related surveys, the difference between our survey and these related surveys, as well as the survey’s main focus, which is shown in Figure 2. Despite the fact that there are several surveys on IoV security [12,65,67,69,71,72,73,74,75], few surveys have covered ML-based solutions in security or in trust schemes in this environment. For example, the surveys in [50,70,76] addressed the use of ML-based trust evaluation in IoV focusing on the trust scheme. In contrast, the surveys in [64,68,77,78] addressed trust in IoV, but ML-based research was not conducted. In the survey in [66], the security aspect was not covered by the authors. In Table 2, we compare previous survey studies in terms of the focused areas of security, trust, and ML approaches as a solution.

## 5. The Concept of IoV

### 5.1. The Internet of Vehicles

The unprecedented developments in computers and communication have escalated the implementation of IoV. The VANET is the previous version of IoV. IoV’s primary objective is to ensure safe driving. While IoV has been embraced by the vehicle manufacturing industry, the technology presents myriad challenges and opportunities that are yet to be explored [75].

Diverse network connections and varying road conditions are considered by intelligent vehicles that are controlled by sophisticated internal software. This software is responsible for managing and controlling vehicle systems. The software depends on information obtained from the interconnected devices and the internet. Most manufacturers decided to adopt IoV applications and start manufacturing their own cars when they discovered the process of converting vehicles from normal mobile nodes to intelligent vehicles. This discovery also brought stiff competition among renowned software-producing companies such as Apple, Google and Huawei [79].

The emergence of IoV technology made it more convenient to own and drive a vehicle. In addition to improving traffic monitoring, the technology aimed to enhance comfortable driving, improve energy management, secure data transmission, and prevent road accidents. While this was good news, the new technology came with many challenges and opportunities. Some of the issues that have still not been addressed are achieving large-scale coverage, exchanging data in a secure environment, managing diverse network connections, and dealing with vehicles with a dynamic topology [80].

### 5.2. Comparison of IoV and VANETs

IoV emerged as a result of the integration of the IoT and VANETs. As such, it is a more advanced version of VANETs. The primary reason for the development of IoV was to strengthen and enhance VANETs’ capabilities. While the two technologies share some similarities in terms of objectives, they also have differences that are worth discussing [65].

**Figure 3:**
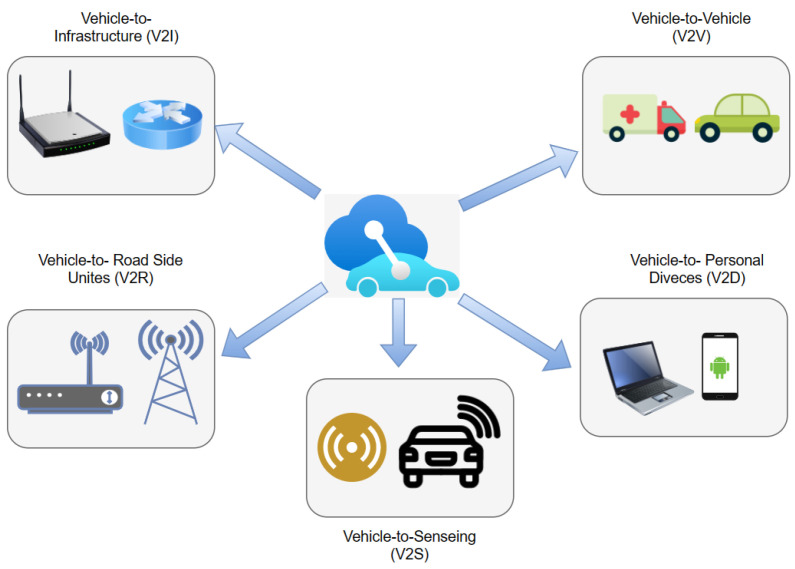
Types of communication used in IoV.

- Goal: Both VANETs and IoV aim to enhance traffic safety and efficiency. However, while VANETs focus more on cost and pollutant emission efficiency, IoV focuses on commercial infotainment. Infotainment is one of the most crucial components of IoV because it helps passengers access services such as online video streaming and file downloading.
- Network specification: IoV has a diverse network framework. The network is used for collaboration and entails communication types such as 4G, Wi-Fi, WAVE, and satellite [81].
- Communication types: IoV has five types of communication, with each type relying on specific wireless communication technology. The five types of communication are vehicle to sensors (V2S), vehicle to road side units (V2R), possible vehicle to vehicle (V2V), vehicle to personal devices (V2P), and vehicle to the infrastructure of cellular networks (V2I) [82]. Figure 3 illustrates the types of communication used in IoV.
- Processing competence: IoV is capable of handling large packets of global data. The system incorporates intelligent computing platforms such as fog computing, cloud computing, and edge computing, which enable it to process large amounts of data at a fast speed [83].
- Compatibility: IoV is easy to use since all the devices used are compatible with the network, thus making it easier for information to be disseminated among the nodes in the most efficient way possible. As such, an interactive environment is created, making it possible to detect hazards early.
- Range of usage: IoV can be used globally; as such, it is an effective technology that can sustain a wide range of applications with different communication and computing capabilities [14,60].
- Network connectivity: Communication is a critical component of IoV networks; for this reason, IoV operates in an environment with the best communication. Moreover, it can easily switch to a stronger and more efficient network in case the current one fails.
- Internet facilities: IoV environments enable vehicles to be connected to the internet at all times. The reliability of IoT networks depends on the speed of the internet and a high bandwidth.
- Cloud computing: Massive quantities of data are processed on a daily basis in an IoV environment. As such, cloud computing is often regarded as the most effective approach for managing vast quantities of data. Cloud computing makes it easier for information to be collected, stored, and analyzed [84].

### 5.3. IoV Architecture

This subsection illustrates the suggestions of numerous researchers about various architectures for IoV. The researcher Liu Nanjie [85] suggested a “Client-Connection-Cloud” system as a three-layer architecture for IoV. The client layer collects data about driving patterns and intra-vehicle and inter-vehicular connections with surrounding vehicles by using all sensors present in the vehicle. The connection layer deals with communication among various units (vehicles, individuals, RSU, and the Internet) in an IoV system to offer vehicle-to-vehicle, vehicle-to-individuals, vehicle-to-RSU, and vehicle-to-Internet communication. In addition, the cloud layer offers services needed for executing tasks, which might not be satisfied by the restrained resources accessible within the vehicle such as mass storage, authentication, and actual-time communication.

Gandotra et al. [86] suggested a three-layer architecture for gadget interaction in which the first layer is utilized for the network region in which gadgets are connected to another device with the network control choosing either wireless or wired interaction. The next layer offers support for IP connectivity and roaming. The last layer backs up the chosen application (IoV and healthcare, among others). Other researchers in [82] proposed a seven-layer architecture: the first layer is the user communication layer, the second is the data acquisition layer, the third is the data filtering and preprocessing layer, the fourth is the business layer, the fifth is the communication layer, the sixth is the management and control layer, and the last layer is the security layer. A transparent connection among the network components is provided by this IoV architecture.

**Figure 4:**
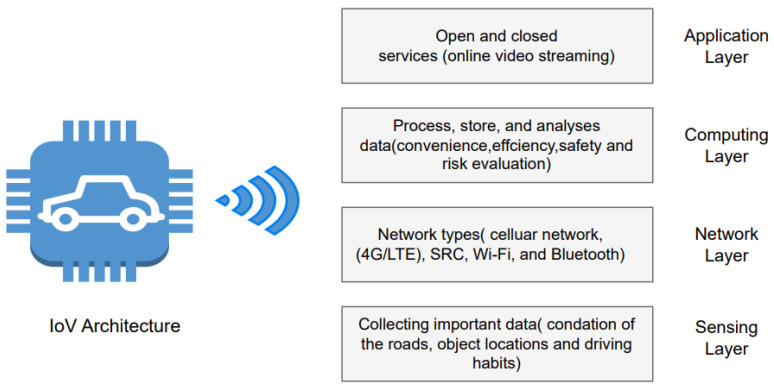
IoV architecture.

IoV structure can be broken down into four main categories: network, computing, sensing, and application, where the sensing layer collects important data about the vehicle’s surrounding environment. The data collected entail information such as the condition of the roads, object locations, and driving habits. The means of data collection is the Radio Frequency Identification Card. The network layer provides all the essential network types such as cellular networks (4G/LTE) and short-range communications (Wi-Fi, Bluetooth) between all the objects in the vehicle. This layer is also connected to the cloud. The computing layer processes, stores, and analyses data required for convenience, efficiency, safety, and risk evaluation. The last layer, the application layer, offers open and closed services. Open services include online video streaming, which is offered by internet service providers. In contrast, closed services are specific IoV applications [12,87,88]. Figure 4 shows the four main categories in IoV architecture.

Different IoV architectures have been suggested by various researchers. Investigation of these architectures illustrates that some of these architectures do not consider security, while some have explained security as services. Since IoV has open network access, the security services provided on these architectures will not be sufficient for securing IoV, which will lead to different vulnerabilities that might affect the entire system. Accordingly, there is a requirement for effective security mechanisms to improve the security of any architecture.

### 5.4. Challenges Facing IoV

In recent years, various technologies have been integrated for the sole purpose of designing and deploying an IoV architecture. Some of these technologies are IEEE 802.11p, Microwave Access, Dedicated Short-Range Communication, Long-Term Evolution, and cloud computing [89]. While IoV is a promising field, it presents myriad challenges. Some of the bottlenecks IoV developers must grapple with include issues associated with data protection, liability, architecture, resource allocation, and diverse interconnected nodes [90,91,92].

The most common IoV network challenges are as follows:

- Complex and diversified networks;
- The security and reliability of services, incompatibility and accuracy of services, and limited storage capacity;
- Data storage and management;
- Scalability;
- Internet provision;
- Poor reception and weak signals that hinder the availability of satellite-based GPS systems;
- Disruptive tolerant communication;
- High mobility of dynamic topologies and vehicle nodes;
- Localization issues;
- Addressing and tracking network fragmentation.

### 5.5. Security Issues in IoV Communication

A middle ground exists between the smart vehicle and the cloud server. This middle ground is commonly referred to as the fog cloud-based IoV. This important component enhances the vehicle’s capabilities by enabling the vehicle to collect and process information locally and to make instant decisions during an accident rather than relying on a cloud server. For instance, several vehicles in the same vicinity can respond to a request from a vehicle within the same area instead of sending a request to the cloud, which may result in a delayed response. IoV highly depends on wireless communication for traffic flow management. For this reason, IoV is susceptible to various attacks such as data disclosure and session key leakage. To address these security issues, the following measures can be employed [75,93,94].

#### 5.5.1. Regular Network Monitoring

Fog systems must be monitored on a regular basis to rid them of any suspicious activities. A fog network can be scanned using either static or dynamic techniques. Typically, a normal network scan involves an antivirus program and a firewall to identify threats and eliminate them to protect the network. Fog environments, however, require more security. Therefore, a new and more effective mechanism for network monitoring is required [95].

#### 5.5.2. Encrypted Data/Communication

Electronic data produced when transmitting between fog and cloud should be protected by encryption. The two most widely used data encryption methods are the Advanced Encryption Standard (AES) algorithm and the Data Encryption Standard (DES) algorithm. The AES algorithm is more appropriate in a fog environment compared to the DES algorithm due to its suitability for sensitive data. Moreover, DES codes are easier to crack compared to AES codes [96].

#### 5.5.3. Authentication and Key Management

During IoV deployment, authentication and key management should be employed between the different devices. Moreover, when authentication and key management are employed between fog servers and cloud servers, they enhance communication in an IoV environment [97].

#### 5.5.4. Wireless Security Protocols

The transmission of sensitive information via mobile phones, wireless cameras, and Radio Frequency Identification (RFID) is common in an IoT environment. As such, for the information to be protected against hackers and other intruders, the wireless communication must be protected. For instance, an unauthorized user can take charge of the network by locking out authorized users, thereby preventing them from using the network. This can be carried out by downloading applications that are irrelevant to the network. Some of the most effective solutions against these kinds of attacks include the use of Wi-Fi-protected access such as WPA2 and WPA3 [98,99].

Now more than ever, IoT networks are vulnerable to cyberattacks. The most common type of attack against IoV involves manipulating and exploiting connections. In other cases, the attackers can gain access to the on-board unit (OBU) and manipulate the data there, affecting functions such as the locking system, emergency brakes, cameras, and wireless system. As such, an effective security mechanism should be capable of protecting the system’s data and nodes from external intrusion. The security of the IoV network is what determines the safety of the driver and passengers in a vehicle. The messages contained in IoV outline accident prevention procedures and methods on how to deal with malicious activity. As such, IoV networks must be protected at all costs due to their sensitivity. To maintain the integrity of the data being transmitted, they must not be altered or modified during transmission [74,77].

### 5.6. Security Threats and Attacks in IoV Environments

Attackers come in different forms. No matter how simple or sophisticated an attacker is, the attack will ultimately affect the integrity of the IoT network. Hence, to protect these networks, it is important to understand the existing threats. Attacks can be categorized into three types: active, passive, and malicious attacks. In active attacks, the attackers can either originate fake messages or alter the information of authentic messages. These attacks are more frequent and difficult to avoid but can easily be detected and are inexpensive to detect. Attackers usually carry out active attacks with the aim of modifying network operations. To prevent this kind of attack, physical security measures should be implemented [100]. The goal of passive attacks is to compromise the target node without changing the content of the transmitted messages. This type of attack is used by attackers to obtain dispersed data from the network. Since passive attacks do not disrupt network operations, they are difficult to detect, and since attackers do not participate in network communication, data encryption can be utilized to prevent such attacks [101]. With malicious attacks, rather than attackers benefiting from the attacks, they are instead initiated with the intent of harming participating nodes. These attacks are dangerous and can cause significant damage. Malicious attackers can even transmit false safety-critical information that puts drivers, passengers, and pedestrians in danger [59,78,102,103,104]. Below is a detailed explanation of some of the attacks to be expected in an IoV environment.

#### 5.6.1. Wormhole Attack

This type of attack is also referred to as a tunnel attack. In this type of attack, the attacker displays the wrong information about its current location to the targeted victim. As a result, the victim node sends its messages through the fake node. Then, all the information received from the sender goes through the attacker node before flowing to the network. Such information is exposed.

#### 5.6.2. Black Hole Attack

A black hole is formed when a vehicle drops packets or refuses to engage in communication. A black hole is akin to an empty space that no one knows exists. The attack starts with an attacker introducing itself as a legitimate node and as a shortcut to a particular destination. Then, the legitimate nodes abandon the route discovery process in favor of the shortcut. The fake node then intercepts the data packets and uses them to create confusion. Eventually, other attacks such as DoS attacks and man-in-the-middle attacks may ensue. In other words, one attack may open doors to other attacks [105].

#### 5.6.3. Dissimulation of a GPS Attack

This refers to the act of intercepting and modifying GPS signals sent by a vehicle within a particular network. These signals are then sent to an unsuspecting receiver after being modified. As a result, the driver and passenger of the targeted vehicle make wrong decisions based on the modified message [60]. In a GPS attack, GPS signals are attacked and the exact location of the vehicle is manipulated. As such, when searching for a particular vehicle, a user may be directed to another location with a fake vehicle. A GPS spoofing attack is a common name for this kind of attack [57].

#### 5.6.4. Denial-of-Service Attack

Every network has a bandwidth within which it operates. Hence, a denial-of-service (DoS) attack feeds a large amount of information to a particular communication channel legally. In this way, the attacker congests the channel and slows it down. While the network is down, the attacker can use the limited resources for illegal business [60]. When communication among legitimate vehicles is jammed, no form of communication can take place. This leads to confusion and panic because these vehicles depend on this communication to make informed decisions such as what routes to avoid [106]. Moreover, information relayed on the network can save lives by preventing accidents from happening. DoS attacks can be categorized into three types [107]:

- Malicious—there is an objective behind the attack;
- Disruptive—the attack has the potential to degrade the network;
- Remote—the attack does not emanate from within the network.

#### 5.6.5. Distributed Denial-of-Service

A distributed denial-of-service (DDoS) attack is a more advanced form of a DoS attack. Unlike a DoS attack, which focuses on specific targets, a DDoS attack is distributive. For this reason, it is more dangerous than a DoS attack. An attack is launched from multiple malicious vehicles to one particular node. The attack can also be launched at different times and locations with the ultimate goal of locking out legitimate users. The fact that the attack can be launched on both vehicles and infrastructure makes it a particularly dangerous type of attack.

#### 5.6.6. Sybil Attack

In this type of attack, the targeted vehicle is made to believe there are vehicles on a particular route, even though the path is clear. As a result, the targeted vehicle takes another route. This attack is made possible by creating multiple fake IDs of a single node, thus indicating that there are multiple nodes in a particular location.

#### 5.6.7. Man-in-the-Middle Attack (MITM)

In this case, the attacker acts as a middleman, but they impersonate both the sender and the receiver. As such, the attacker intercepts messages from both sides and sends wrong information to unsuspecting nodes. This type of attack can be either active or passive. This is one of the most dangerous types of attack because the attacker can cause massive damage, even with minimal information about the network. This type of attacker often targets roadside unit nodes because they are responsible for providing services by sending messages, updates, and resources to handle all communication requests.

#### 5.6.8. Masquerading Attack

To masquerade is to pretend. As such, the attacker pretends to be someone else by hiding their true identity. Once that is achieved, the attacker can then act as a legitimate node with the sole intention of producing misleading messages or modifying the received messages. For instance, an attacker may receive a message indicating that the road is clear. In contrast, the attacker broadcasts that there is an accident ahead. As a result, road users may decide to take an alternative route or slow down. This type of attack disrupts traffic and may create a huge traffic jam [108].

#### 5.6.9. Eavesdropping Attack

In this case, the attacker gains access to the network from the outside. Even though they are not an active participant in the network, the attacker is able to obtain private confidential information illegally and use it for personal gain.

#### 5.6.10. Malware Attack

In this type of attack, malicious worms or viruses are transferred from one point to another through electronic files. The files can then infect the network instantly or gradually [109].

## 6. Solutions for Security and Trust in IoV

**Figure 5:**
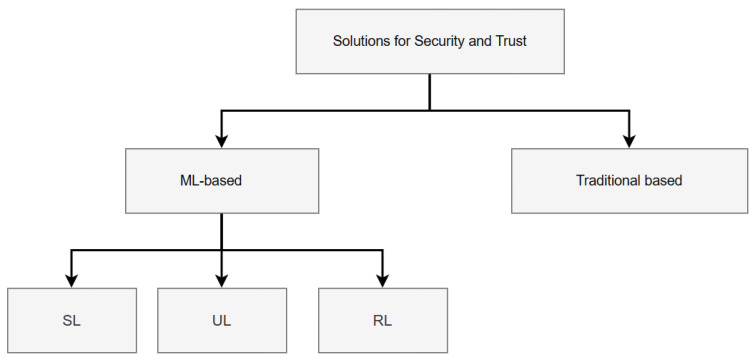
Classification of security and trust solutions based on traditional and machine learning (ML)-based approaches.

As depicted in Figure 5, this section classifies security and trust solutions based on traditional and ML-based approaches.

### 6.1. Traditional-Based Solutions

Over the years, trust management has become a popular method for maintaining secure and reliable vehicle networks. Traditional solutions, such as Bayesian-inference-based, cryptography-based, blockchain-based, and fuzzy-logic-based trust management techniques can all be used in vehicular networks. Conventional trust models are also known as traditional trust management models, and they do not require any advanced data analysis or statistical inference methods to work. In [110], the MARINE system, which was presented by Ahmad et al., is used to detect malicious vehicles, which are vehicles capable of launching MITM attacks and canceling credentials. Furthermore, the MARINE system is an example of a hybrid model that takes into account the possibility that a trustworthy vehicle may send a false message due to faulty hardware as well as the likelihood that a malicious vehicle may send real messages. Data trust, node trust, infrastructure-to-vehicle trust, and vehicle-to-vehicle trust are often taken into account in this proposed paradigm. The trustworthiness of a node is determined by the sum of all prior connections with the target vehicle and neighbor opinions. On the other hand, data trust considers the quality of data received, the vehicle’s ability to transfer messages, and surrounding entities’ recommendations. In most cases, calculating vehicle-to-vehicle trust requires assigning each vehicle the responsibility of writing a good report about an honest vehicle and a negative report with information about a dishonest vehicle. After the reports are generated, they are sent to the roadside unit, which is where the infrastructure-to-vehicle report is generated and updated. Three attack scenarios were used to test the suggested MARINE model. It makes use of SUMO (urban mobility simulation) as well as a cars in network simulation (VEINS) [111,112,113].

Bayesian-inference-based trust models employ Bayesian theory, which addresses the uncertainty of data-centric modeling and inference through the application of probability and statistics. While considering both regional and international vehicle trust, Zhang et al. proposed a trust management approach based on the TrustRank algorithm [114]. To compute local trust, the Bayesian inference model is applied to previous vehicle interactions, and once local trust is computed, a trust link graph is constructed. Global trust is calculated by combining social factors based on the driver and vehicle, and before applying the TrustRank algorithm, the behavior with local trust values and previous global trust values is considered. The PageRank algorithm determines the most trustworthy automobiles as seed vehicles. True negative and true positive performance indicators are used to evaluate this strategy. Furthermore, these models are simulated with the help of VEINS simulation. During its evaluation, this model took into account newcomer attacks, on–off attacks, and collusion attacks.

The fuzzy-logic-based model takes into account the imprecision of human reasoning when making decisions in uncertain and imprecise contexts. In [115], Guleng et al. proposed a decentralized architecture that integrates a vehicle’s own experience and peer recommendations using fuzzy logic. This is carried out with the intention of labeling the vehicle’s unintentional dishonest behavior. The concept of RL is enforced by an indirect trust score for vehicles with no direct connection to the trustor. In measuring direct trust before using fuzzy logic, the percentage of authentic messages passed by the vehicle and the proportion of all messages conveyed by the vehicle are taken into account. Q-learning is used to gather feedback on the target vehicle from nearby vehicles, which is then used to calculate indirect trust. The simulations for this model were carried out with the help of a network simulator (NS-2.34) [116].

In the cryptography-based trust model, the emphasis is on encrypting data to increase secrecy and prevent unauthorized users from decoding the data. In [109], Gai et al developed a concept in which one inquirer uses cookies to rate the services offered by another vehicle. These vehicles are signed by a certificate authority to prevent counterfeiting by other vehicles. When a vehicle is presented with a request from another vehicle, it transmits its cookies along with the specific service that is needed, and the vehicle that receives the request uses this information to assess the service provider’s trustworthiness. In this paradigm, cookies in the requester’s record are used to calculate direct trust, which is based on previous encounters. Indirect trust, on the other hand, is calculated using the service provider’s shared cookies. VANETism was used to run simulations of this model. The most common authentication attacks include wormhole attacks, GPS deception, secrecy attacks, and routing attacks [67]. As presented in [58], for an IoV architecture to be considered secure, it must have low error tolerance, high mobility of IoV entities, private routing of data, and key distribution management. Common countermeasures for the above-mentioned attacks include honeypot, threat modeling, and intrusion detection systems. In the honeypot approach, the system deceives attackers and identifies malicious actions in architecture. In threat modeling, Petri net modeling is used to authenticate, control, and model complex vehicular networks. Crowdsourced data must be authenticated to deal with malicious attacks that often mislead the vehicle and force it to take a different route than the desired one. These messages must be detected in time so they can be eliminated before causing any damage. In [117], the authors emphasize the importance of authorizing and verifying information. By doing so, the collected information will be under supervision, thus preventing the vehicle from being misled. The other commonly used method is scalable privacy. An individual’s privacy is protected, and the individual is given the alternative to control the flow of information. In this case, privacy is sacrificed for privacy. Scalable privacy ensures that data are extracted from a big group, thus making it difficult for the owner’s data to be distorted. Sybil attacks violate the safety of passengers. These attacks introduce nodes that assume multiple identities, thus disseminating false messages in the network. The authors provide insights into the mechanism of understanding by proposing a comprehensive heterogeneous communication technology architecture for secure and private vehicular clouds that serve IoVs. This type of attack can cause confusion and is responsible for most of the accidents in IoVs [67]. The authors in [118] created a Sybil detection method known as Voiceprint that is based on signal strength indicators. Voiceprint compares all the received time series, which is unlike the other common methods used to prevent Sybil attacks. Moreover, Voiceprint can be enhanced to carry out detection on service channels. This enhancement decreases the false positive rate. GPS Spoofing is a type of attack that focuses on Global Positioning System signals. It misleads these signals, which places passengers in danger. In [119], it is shown that the best way to address this type of attack is to use a two-factor authentication system that uses digital signatures and time synchronization to prevent spoofing. The hash signals are encrypted to further protect the system. Detecting masquerading attacks is not an easy task. The authors effectively address VANET vulnerabilities and highlight the complex difficulty of preventing masquerading attacks in autonomous navigation systems. In [120], to address this problem, network specialists recommend an anomaly detection model that detects attacks using signal strength fluctuations. The malicious nodes are detected by factoring in the maximum speed of the nodes and the time of reception. Data falsification attack detection mechanisms have received increasing attention from security analysts. The authors present a hardware-independent technique for detecting masquerade attacks that is based on an adaptive anomaly detection model that takes signal strength variations into account. Furthermore, this methodology emphasizes the criticality of employing adaptive and efficient threat detection systems. In [121], the main idea behind these mechanisms is to improve IoV network throughput and ensure accurate information relay to the neighbors. At the same time [122], hierarchical temporal memory can be employed to mitigate inconsistencies in the communication medium. In [123], to improve the security of smart vehicles, a two-level cryptography authentication technique is used.

The methods used to address common attacks can be categorized in terms of availability, data integrity, authentication, and confidentiality [124]. Availability, which refers to the overall network uptime, is a crucial element in IoV. Given that vehicles depend on traffic to make certain decisions, availability must be considered when selecting the best solution to common threats. A lack of availability can lead to the total shutdown of an IoV environment. Some of the most common attacks against availability are DoS, jamming, and DDoS attacks. In [125], to detect malicious nodes that affect the availability of vehicular networks, an algorithm for DoS attacks is proposed by detecting the sending of irrelevant packets by malicious nodes. Data integrity refers to the incorruptible nature of the information distributed in an IoV environment. In other words, the data received and sent should be the same. The most common type of attack against data integrity is the man-in-the-middle attack [126]. In [110], the use of trust models to identify malicious nodes is recommended. Once the malicious nodes have been identified, their credentials are revoked, thereby stopping the man-in-the-middle attack in its tracks. Confidentiality refers to the nondisclosure of private information pertaining to the vehicle as well as the user. Hackers are always on the lookout for loopholes they can exploit to access confidential information and use it for sinister motives. For this reason, measures are put in place to ensure that unauthorized individuals cannot access critical information such as the location and routes of the vehicle. The most recognized type of attack against IoV is known as eavesdropping. In [127], network specialists use models that generate false traffic packets to mislead attackers. The false data packets shield roadside unit (RSU) hotspots from unwarranted intrusions. Additionally, a real-time monitoring system for road conditions can be used to prevent collision attacks, thus protecting confidential information from potential attackers [128]. Lastly, authentication refers to the verification of user identities or system identities in an IoV environment. The most common type of attack against authentication is the Sybil attack. This type of attack can be addressed by grouping nodes into various zones and eliminating the malicious nodes [129].

### 6.2. Machine Learning-Based Solutions for Security

The massive production and use of digital gadgets has led to tremendous growth in IoT usage. The IoT is currently being used in industries, transport systems, power systems, and agriculture, among other domains; this growth in usage, however, has also brought about myriad security problems. The IoT uses a variety of advanced technologies such as Bluetooth, RFIDs, wireless sensor networks, cloud computing, and Zigbee, among many other systems. As such, it is challenging to protect all these networks against malware attacks, eavesdropping, DoS attacks, and virus injection [76,130]. ML has developed as a security-enhancing technology in recent years. A growing number of studies has established that there has been an increase in ML-based contributions to network security over the past few years. The five most frequently explored security fields are software protection, malware detection, reaction policy, game protection, and biometric authentication. Of all these fields, malware/intrusion detection is the most heavily researched. Moreover, autonomous vehicles and wireless networks are among the most frequently addressed targets of attacks. The IoT connects millions of devices. These devices are dynamic and result in a complex network. It is for this reason that ML techniques are used for detecting intrusions, CPS attack detection, malware detection, privacy maintenance, and trust. These elaborate techniques are ineffective, however, in performing dynamic responses as far as the IoT is concerned [131,132,133,134]. In this survey, ML-based trust management schemes and security solutions are classified. We present trust and security solutions for IoV environments, and we classify them based on the three types of ML models: supervised learning, unsupervised learning, and RL.

#### 6.2.1. Supervised-Learning-Based Solutions

Gyawali and Qian describe an ML-based framework that uses fake alarm messages and positional falsification to detect vehicle misbehavior. By communicating information via a beacon, the vehicles keep each other informed about various events such as road conditions, accidents, emergency vehicles, and collision warnings. The authors of this work used beacons and information deviations from standard protocol conditions as input to ML algorithms. This study compared five different types of ML algorithms, with the decision-tree classifier achieving the greatest accuracy of 95% [135]. In [136], the authors proposed using k-nearest neighbors (kNN)and support vector machine models to detect malicious node attacks in VANET. By adapting KNN models to attack detectors, network features such as IP addresses, delays, jitters, dropped packets, and throughput are learned. The authors used an NS-3 network simulator to generate a multihop communication scenario, and the result showed 99% accuracy. In [137], the authors proposed a scheme to detect jamming attacks in a vehicular network. Recent work developed a data-centric misbehavior detection system for IoVs. This research is unique in that it combines plausibility tests with traditional supervised ML algorithms to improve detection accuracy. The authors evaluated the performance of six supervised ML algorithms using location and movement plausibility checks. With the added plausibility checks, the findings reveal a 5% and 2% gain in precision and recall, respectively [138]. In IoV networks, real-time data integrity is dependent upon the detection of data falsification. The IoT and ML are used to address vehicular network (VN) security challenges in [139]. The study addresses backdoor, DDoS, and MITM attacks and uses TON-IoT dataset ML algorithms for intrusion detection. Intrusion detection uses RF, NB, and KNN machine learning. KNN has the highest accuracy, demonstrating ML’s ability to detect VN attacks. In [140], the authors proposed an IDS scheme to classify normal and malicious traffic messages in vehicle networks using eight supervised models. Their scheme demonstrated high performance. The authors in [141] proposed a Randomized Search Optimization Ensemble-based Falsification Detection Scheme (RSO-FDS) that uses Random Forest (RF) as its primary model. This scheme’s efficacy in detecting falsification attacks is demonstrated by the performance evaluation and addresses the detection of falsification, which is necessary to maintain trust in the data shared between vehicles and IoV services. These studies provided a foundation for enhancing the security of vehicular networks and IoV with supervised machine learning. Future research should concentrate on addressing all of these obstacles and expanding the applicability of these techniques to situations in the real world.

#### 6.2.2. Unsupervised-Learning-Based Solutions

The authors in [142] proposed an approach to defend against DoS attacks. The unsupervised deep learning choice was Deep Contractive Autoencoders (DCAEs). They compared actual and predicted data depending on mean square error and mean absolute error metrics. Angelo et al. developed a data-driven strategy for detecting DoS assaults as well as three additional types of in-vehicle network threats. Unsupervised learning is used to extract relevant features from data linked to the controller area network (CAN) bus. It reflects CAN behavior based on those traits, and any divergence from the learned behavior is regarded as a system attack. Additionally, by observing associated CAN bus metrics, this work adopts a data-centric scheme to narrow down the type of attack. For a car-hacking dataset, the suggested scheme’s performance evaluation findings demonstrate a high performance [143]. The authors in [144] addressed the significant issue of DoS attacks in vehicular networks and investigated the viability of using unsupervised learning algorithms for DoS detection. Since DoS attacks pose threats to connected car functionality and safety, their detection and mitigation are of utmost importance. Future research should focus on enhancing the accuracy and adaptability of these algorithms, evaluating their performance in real-world scenarios, and developing hybrid approaches that combine unsupervised and supervised learning techniques. Detection of Anomalous Behavior in Smart Conveyance Operations (DAMASCO) is a security system described in Reference [145], which discusses the security challenges in VANETs and presents a system called DAMASCO. The system detects anomalies in vehicle-to-vehicle (V2V) communication using a statistical approach. The findings prove that the system is capable of identifying potentially malicious nodes while maintaining a low false positive rate. These studies show that unsupervised learning approaches can improve vehicle network security, notably by identifying DoS attacks. UL can improve the security and dependability of connected vehicles and IoT-driven transportation ecosystems by tackling the difficulties in these references and refining unsupervised learning algorithms. This adoption of unsupervised learning signifies a significant advance in enhancing the integrity and safety of current transportation systems.

#### 6.2.3. RL-Based Solutions

RL-based spoofing attack detection can be used to prevent jamming attacks. In this approach, RSSI is used to find spoofing data. Q-learning makes the final decision. There is no need for prior information about the network model and the attack model [146]. The threshold of an IoT attack detection agent can be established through interaction with the surrounding environment. It should be noted that RL is more suitable for specific network scenarios [147]. For instance, an RL-based scheduler is preferred because of its ability to adapt to various types of traffic and reward functions [148]. In addition, deep RL can be used for signal authentication [149]. In other cases, a deep RL detection system can be used to enhance the security of an IoT system [150]. Still, there is room for further improvement in terms of research work on the characteristics of intrusion. The agent observes the attack detection rate as well as the false alarm rate and then takes the required action. The reward of the process is immediate. The selection of a new optimal policy will update the system reward with new detection and false alarm rates. What is more, the information acquired in the observation, such as the immediate reward, can be used to adjust the agent’s policy. This process is repetitive [147]. One of the best approaches for IoV network protection is analyzing packets of data to detect any potential vulnerability. This approach utilizes a cluster-based topology in which packets are captured and analyzed through fuzzy logic. What is more, Q-learning is used to detect DDoS attacks [151]. To prevent jamming attacks, an RL attack algorithm is used to perform Q-learning so as to study past actions. It has been established that a well-learned network boosts the optimum performance of the transmitter in the case of a jamming attack [152]. In [153], the authors presented an approach based on RL to identify and address the transmission of false or inaccurate data by malicious vehicles, posing a risk to road safety. This methodology uses RL models to analyze V2X data, effectively classifying incoming data as either legitimate or showing malicious behavior in a timely manner. The research addresses the increasing significance of securing V2X communication in the era of connected vehicles and establishes the stage for more advanced misbehavior detection mechanisms, significantly enhancing the safety and efficacy of IoV networks. The authors in [154] proposed an attack-resistant framework for optimal service placement using deep reinforcement learning (DRL) and integer linear programming (ILP) models. In a dynamic IoV environment, this innovative strategy optimizes service placement to minimize latency while efficiently utilizing limited edge resources. Secondary mapping and service recovery mechanisms are also introduced to mitigate edge attacks and failures. This design improves the user experience, service latency, resource use, and active edge nodes. It may improve IoV network stability and security. These references highlighted the significance of utilizing innovative approaches, such as reinforcement learning techniques, to address security challenges in connected vehicular and IoT networks. They presented valuable insights for enhancing detection, authentication, and attack resilience in these environments. The security solutions based on ML are shown in Table 3.

### 6.3. ML-Based Solutions for Trust

#### 6.3.1. Supervised-Learning-Based Solutions

In [155], an ML-based trust model is developed to extract relevant features from the vehicular network. The authors employ a Bayesian neural network (BNN) for the classification process. The model had high-performance prediction and classification accuracy. In [156], the authors proposed an M2M vehicle-based ML network (MLN)-based trust to detect suspicious activity. The intense recharge of the battery (XGBoost) method is used, and the system has entropy-dependent data augmentation combined with this approach. The effectiveness of this method is measured based on XGBoost and Random Forest, and the results showed increased efficiency with 10% inaccuracy. In [157], the authors proposed an ML technique to detect fake position attacks in VANETs. The applied technique is KNN, which is a classification algorithm under supervised learning. The direct trust and the trust metric are calculated depending on the data exchanged by the nodes of the network. The kNN classification and trust model metrics can detect misbehaving nodes using receiver power coherency. A random forest algorithm attack classification is proposed in [158]. Intrusion detection system (IDS) classifiers are used for each vehicle depending on a random forest algorithm, and each vehicle shares its knowledge with other vehicles. The value of this algorithm is generated by the trust factor of the received classifier. Over four types of attacks are classified with an F1 score of 97% and a 4% false-positive rate by using a network security laboratory-knowledge discovery data mining (NSL-KDD) dataset. In [159], the authors proposed a classification-based trust model (CTM) for IoV to improve the security of the communication environment. They used an ML model to indicate the vehicles as trusted or untrusted, which is a Naïve Bayes model. The model gave a good answer for both the different kinds of vehicles and the trust factor. The authors in [160] focused on identifying potential Sybil vehicles and protecting messages from Sybil attacks. The proposed strategy integrates metaheuristic methods during the establishment of communication to identify possible Sybil nodes and employs trust certification mechanisms to guarantee the integrity of messages. The importance of employing a variety of methods to properly prevent Sybil attacks is highlighted. The author in [161] investigated the expanding field of IoV and the need for trust assessment schemes to defeat insider assailants. For accurate trust assessment, precise weight assignment and the definition of a minimum acceptable trust threshold are essential. The paper employs an IoT dataset, adapted from CRAWDAD to an IoV format, comprising information on 18,226 interactions among 76 nodes, both honest and dishonest. It computes important parameters, including packet delivery ratio, familiarity, timeliness, and interaction frequency. Two feature matrices are generated: FM1, which takes individual parameters into account as features, and FM2, which averages pairwise computations for each parameter. Once the truth has been established by unsupervised learning, supervised machine learning can be employed for categorization. The results demonstrate that FM2 is superior at accurately classifying dishonest vehicles. These references enhance trust and security techniques in vehicular communication networks, addressing important difficulties and making VANETs and IoV systems safer and more reliable. These research will likely develop and enhance these models to address evolving threats.

#### 6.3.2. Unsupervised-Learning-Based Solutions

Kamel et al. introduced a generic RNN-based approach for the global detection of Sybil attacks. This study investigates the four distinct consequences of a Sybil attack: traffic congestion, data replay, DoS random, and DoS disruptive. OBUs and RSUs identify misbehavior in vehicles in the first stage. They notify the misbehavior authority (MA) of any malicious activities by sending misbehavior reports (MBRs). The objective of the MA is to obtain a broad picture of what is going on in the world of vehicular nodes. The MA carries out eleven different information checks and feeds these as input into an LSTM-based RNN network that detects the correct type of Sybil attacks. The authors also use an autoencoder algorithm to enforce feature compression in their work. They use the OMNET++ simulator to analyze the performance of their suggested model, which has a 95% accuracy [162]. In [163], Tangade et al. suggested a trust model that uses deep neural network models (DNN) to improve dependability while simultaneously lowering latency. Each car in this model communicates with the vehicles around it, and this communication is utilized to award points to each vehicle. Furthermore, the awarding of points to cars is used to classify communications as either honest or dishonest, and it is subsequently used in the computation of trust scores using deep neural networks. The message generated by the vehicle is transmitted and received by the roadside unit, which authenticates the source before using deep neural networks to determine reward points based on the driver’s behavior. Using deep neural networks, the received message can be categorized as either honest or dishonest, and the mediator’s trusted authority then calculates the vehicles’ updated trust score. The TensorFlow simulator and a network simulator (NS-3) were used to test this model. In [164], Siddiqui et al. suggested another trust model that uses ML to identify rogue vehicles in the vehicular network. Similarity, familiarity, and packet delivery ratio are the three trust factors used in this model. This model clusters the data for label assignment using multiple unsupervised learning algorithms. Various supervised learning techniques are used to classify cars as either honest or dishonest, and they also aid in the acquisition of an ideal threshold. MATLAB was used to run simulations of the suggested trust model. The authors in [142] proposed an approach to defend against DoS attacks. The unsupervised deep learning choice is Deep Contractive Autoencoders. Using mean square error and mean absolute error, they compare the real data with what was predicted. The authors [165] presented a trust management strategy for VANETs that relies on machine learning and active detection technology. The evaluation process assesses the trustworthiness of vehicles and events in order to assure the reliability of communication. The active detection method improves the filtering of malicious nodes, while the Bayesian classifier identifies malicious vehicles. These references help to develop trust mechanisms in vehicle networks, and future studies will concentrate on their practical application, model improvement, and research of novel technologies to address new challenges.

#### 6.3.3. Reinforcement-Learning-Based Solutions

In [166], the authors presented a proposal detailing their previous trust evaluation method; this is another method that amalgamates the Q-learning algorithm and fuzzy logic. Incorporating trust’s incertitude and vagueness is beneficial. The computation of direct trust values is performed first through the use of a Q-learning algorithm, with fuzzification of the indirect trust value, average time delay, and direct trust value being the second step. They then obtained the final result by using the inference rules in a fuzzy logic system. Simulation experiments were carried out in dissimilar settings. One can assimilate from the experimental results that, in addition to this method supporting context awareness, it is capable of resisting attacks and enhancing accuracy. However, the method’s support for subjectivity was ambiguous, and data privacy protection measures were absent. An RL-based obfuscation scheme helps enhance the privacy of the vehicle. The vehicle initiates communication with the RSU, indicating its location coordinates and semantic location. The information is used to assess the privacy level of the vehicle, its current state, and its real location to update the Q-function. This approach’s main objective is to minimize the vehicle’s privacy gain [167]. In [168], the TROVE model is proposed to authenticate and evaluate the trust value of the sender. The authors created an RL model to set the evaluation strategy for vehicles and decide how much to trust the current evaluation strategy based on the information available. In [169], the authors propose an indirect reciprocal incentive mechanism for VANETs. It attempts to reduce OBU attacks motivated solely by self-interest by encouraging OBU cooperation. DRL is used to reduce the motivation for attacks and make informed decisions. Simulations demonstrate superior performance in comparison to conventional strategies. These references are intended to resolve emerging challenges, improve the performance of trust mechanisms, and contribute to the practical implementation and RL-proposed solutions for vehicular networks. The trust solutions based on ML are shown in Table 4.

**Figure 6:**
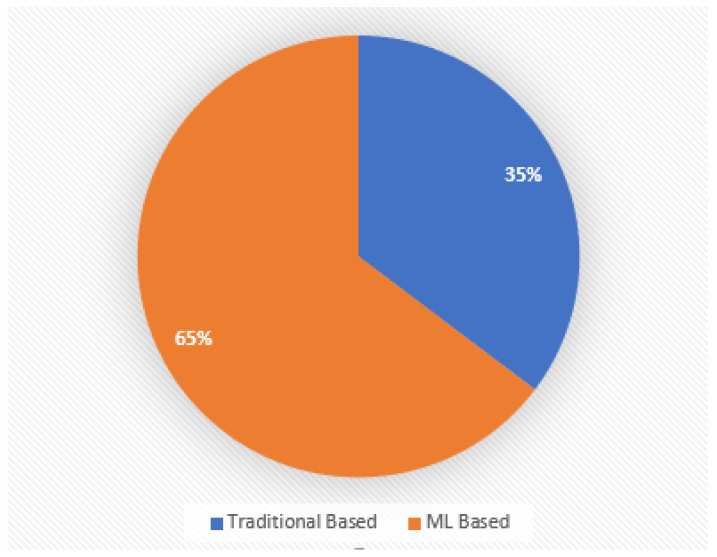
Percentage of solutions based on traditional and machine-learning-based approaches.

**Figure 7:**
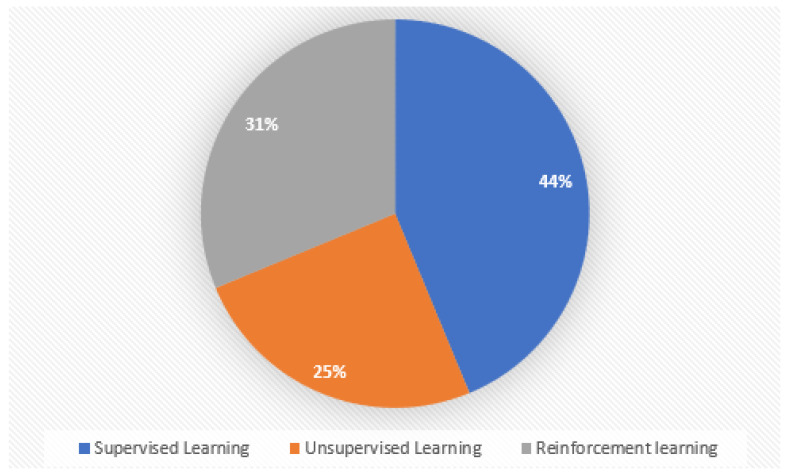
Percentage of solutions based on three types of ML-based approaches.

Various researchers have proposed numerous security solutions and trust schemes for securing and establishing trust in IoV. Figure 6 shows that ML-based solutions are more widely used than traditional solutions for detecting malicious nodes and attacks in IoV networks, and they are widely used to improve trust and security. The percentage of each ML-type solution is depicted in Figure 7. The objective of supervised learning is to predict training data dependencies and find the solution to the issue presented in the data. Unsupervised learning utilizes unlabeled data to recognize patterns. RL improves efficiency monitoring and provides real-time data on the performance of an advanced system. RL uses trial and error to test a model in order to learn it. Data collection in certain IoT contexts can be a problem. RL is able to produce its own dataset [76,170]. ML plays a critical role in helping autonomous vehicles make informed decisions. Despite its strengths, ML faces a range of challenges such as failure in the detection of attacks, wrongly classified objects, the recognition of driver monitoring patterns, vehicle theft, and compromised functional safety [57,76,171,172].

### 6.4. Future Directions

There are numerous potential future avenues for addressing emerging challenges, improving the performance of trust and security techniques, and contributing to the practical implementation and development of provided solutions in vehicle networks.

#### 6.4.1. Real-Time Testing

Testing in the actual world would be beneficial to extend the experiments to IoV environments in the real world. Testing in the field can provide information about the applicability of machine-learning-based security solutions in the real world and their effectiveness in real-world vehicular networks [139,141,142,143,144]. Researchers can refine and optimize algorithms to ensure reliability in real-world scenarios. Real-time testing also informs adaptive models that learn and adapt to changing security threats. This iterative technique improves ML-based security solutions for real-world vehicular networks.

#### 6.4.2. Unknown Attacks

It is essential to consider new or unknown attack types. The development of adaptive machine learning models that can detect new attack patterns and constantly changing threats to security in vehicle networks is a topic that could potentially benefit from further exploration in the future [135,138]. In vehicle networks, the proactive detection of unknown intrusions is a complex challenge requiring innovative approaches. For future research, it is worth considering investing in the development of adaptive machine learning models. This might lead to the development of security systems that are more resilient and adaptive, able to handle developing threats in the connected vehicle environment.

#### 6.4.3. Blockchains

In most IoV scenarios, blockchains can offer a wide variety of novel solutions. The majority of IoV scenarios involve the generation and interchange of a significant amount of data, and the majority of conventional technologies are not suited for efficient utilization in these types of scenarios. As blockchain research is still in its infancy, however, the blockchain could enhance IoV’s trust, security, and privacy [165]. The use of blockchain technology in IoV has great potential for protecting privacy. This is due to its privacy-focused characteristics and the decentralized management of data. Future research should focus on resolving issues with scalability, performance, and privacy to fully utilize these advantages. It is essential to overcome these challenges to develop a secure and reliable IoV environment.

#### 6.4.4. IoV and Big Data

Since the IoT is expanding and huge amounts of data are being produced in vehicle networks, researchers may investigate how these two technologies might be combined in the future to enhance trust models and security solutions. Researchers may obtain insights from vast amounts of data using IoV and Big Data, improving trust models and security in connected vehicle networks. Big data analytics can improve vehicle real-time communication reliability by developing adaptive trust mechanisms. By employing machine learning to identify anomalies and potential intrusions, this combination helps provide proactive security solutions. Combining IoV with big data could lead to more secure, efficient, and reliable intelligent transportation systems.

#### 6.4.5. Availability of Datasets

In order to further study and compare machine-learning-based security solutions in IoV environments, it is essential to create and share open datasets that represent real-world vehicular network scenarios [136,138].

## 7. Conclusions

The Internet of Vehicles presents a promising technology aimed at enhancing driving comfort, improving energy management, securing data transmission, and preventing road accidents. However, these advantages are accompanied by significant challenges, particularly in the domains of security and trust. This survey discusses the critical role of ML as a potent solution to address security concerns and trust management in an IoV environment. We presented an overview of IoV and trust management, discussing security requirements, challenges, and attacks. Additionally, we introduced a classification scheme for ML techniques and surveyed IoV ML-based security and trust management schemes. This survey highlights the significant role that ML technology can play in providing a secure environment for the operations of IoV. Through an extensive study of diverse machine learning methodologies and their practical implementations, such as supervised learning, unsupervised learning, and reinforcement learning, we demonstrate the capability of machine learning to effectively address security and trust challenges in IoV. ML technology is increasingly recognized as an effective method for addressing the challenges posed by malicious nodes and attacks on an IoV network when compared to conventional methods. In order to protect the safety and security of drivers and users within IoV dynamic and interconnected environments, it is crucial to adopt modern technologies such as machine learning. To improve trust and security, future work should focus on real-time testing, resilience against unknown attacks, blockchain integration, IoV and big data management, and expanded dataset availability.

## Figures and Tables

**Table 1:** Trust properties.

Trust Properties	Description
Direct	This attribute calculates trust value based on trustor–trustee relationships.
Indirect	Trust value is calculated from the suggestions and opinions of the trustor’s various neighbors.
Subjective	Trust value is calculated from the personal views of the trustor.
Objective	The parameters of the observed trustee entity are what determine the value of trust, which is derived from those parameters.
Local	The value of trust is exclusively accessible to the trustor and trustee and cannot be accessed by other users in the network.
Global	Each entity that is part of the network has its own trust value that is known by every other entity.
Asymmetric	This is when one entity gives trust to a second entity but the second entity does not give trust to the first entity.
History-dependent	Trust value is given depending on the previous behaviors of the entity under observation.
Context-dependent	The importance of trust depends on the surrounding circumstances.
Composite	Trust value is based upon various parameters.
Dynamic	If the initial trust value was generated with certain parameters and those values later change, the trust value will also change.

**Table 2:** Comparison of surveys in terms of the focused areas of security, trust, and ML approaches.

Citation	Year	IoV Security	IoV Trust	Machine Learning	Comparison
[12]	2017	+			The survey does not explore ML techniques and the trust scheme.
[74]	2017	+			The survey discusses only security challenges and the trust and ML-based solutions are not explored.
[64]	2018	+	+		Only three types of trust models are considered. The survey does not discuss ML-based solutions.
[77]	2018	+	+		The survey does not provide a number of solutions based on ML techniques for security or trust in IoV.
[78]	2018	+	+		The authors present cryptographical solutions and ML-based solutions are missing.
[65]	2019	+			The authors present many security solutions but detailed information on trust and ML techniques are missing.
[66]	2020		+	+	There is no security challenge discussed in this survey.
[76]	2020	+		+	The survey discussed ML-based solutions for security but not for trust.
[67]	2020	+			The survey discusses only security protection but the trust and ML-based solutions are not presented.
[75]	2020	+			The survey is missing the trust scheme and ML-based solutions in IoV.
[68]	2020	+	+		The survey does not discuss ML-based solutions for security and trust.
[69]	2021	+			The survey is missing the trust scheme and ML-based solutions in IoV.
[70]	2021	+		+	Details about using ML-based solutions in the trust problem are missing.
[50]	2022	+		+	The authors do not explore the trust challenge and its solutions.
[71]	2022	+			The survey does not explore ML and trust.
[72]	2022	+			The survey does not mention ML or trust.
[73]	2022	+			The authors present the security challenges of IoV environments, but ML-based solutions and trust are missing.
Our Survey	2023	+	+	+	Our survey focuses on the areas of security, trust, and ML approaches.

**Table 3:** Security solutions based on ML.

Citation	Year	Focused Area	Solution Technique	ML Type	Algorithm	Attack Type	Object
[135]	2019	Security	Machine Learning	SL	Decision-tree classifier	Vehicle misbehavior	Detect vehicle misbehavior
[136]	2019			SL	KNN and SVM	Malicious node attacks	Detect malicious node
[137]	2019			SL	CatBoost	Jamming attacks	Detect jamming attacks
[138]	2020			SL	Plausibility checks and traditional SL	A data-centric misbehavior	Misbehavior detection system for IoVs
[139]	2022			SL	RF, NB, and KNN	Backdoor, DDoS, and MITM attacks	To detect and mitigate various IoV attacks using ML algorithms
[140]	2022			SL	Eight SL models	Malicious messages	Classification of normal and malicious messages in vehicle network
[141]	2023			SL	RF	Falsification attacks	To protect IoV data, identify and prevent falsification attacks.
[142]	2019			UL	DCAEs	DoS attacks	Defend against DoS attacks
[143]	2020			UL	UL	Four types of attacks	Detect DoS attacks and three other types of attacks
[144]	2022			UL	K-Means, Gaussian Mixture, and Dbscan Clustering	DoS attack	To identify and mitigate DoS attacks that compromise connected vehicle function and safety
[145]	2023			UL	Median Absolute Deviation	Anomalies in V2V communication	To detect malicious nodes with low false-positive rates
[146]	2018			RL	Q-learning	Spoofing attack	Find spoofing data
[149]	2019			RL	DRL	Malicious node attacks	Signal authentication
[151]	2019			RL	Q-learning	DDoS attacks	Detect DDoS attacks
[152]	2019			RL	Q-learning	Jamming attack	Prevent jamming attack
[153]	2022			RL	Q-learning	Malicious data transmission in V2X communication	Classifying incoming data as legitimate or malicious improves security
[154]	2023			RL	DRL and ILP	Edge attacks	To improve network stability and enhancing security mechanisms

**Table 4:** Trust solutions based on ML.

Citation	Year	Focused Area	Solution Technique	ML Type	Algorithm	Attack Type	Object
[155]	2018	Trust	Machine Learning	SL	Bayesian neural network	Malicious node attacks	Extract relevant features from the vehicular network for a trust model
[156]	2019			SL	XGBoost and RF	Suspicious activity	Detect suspicious activity
[157]	2020			SL	KNN	Fake position attacks	Detect misbehaving nodes
[158]	2020			SL	RF	Four types of attacks	Generated the trust factor
[159]	2020			SL	NB	Malicious node attacks	Indicate the vehicles as trusted or untrusted
[160]	2022			SL	Metaheuristic	Sybil attacks	To identify Sybil nodes and protect messages from Sybil attacks.
[161]	2023			SL	KNN and SVM	Insider attacks in IoV	By computing parameters and identifying dishonest vehicles, the aim is to accurately evaluate trust
[162]	2019			UL	RNN-based	Sybil attacks	The global detection of Sybil attacks
[163]	2019			UL	DNN	Dishonest vehicle	Classify communications as either honest or dishonest
[164]	2019			UL	Various UL models	Dishonest vehicle	Classify the cars as either honest or dishonest
[165]	2022			UL	Bayesian and active detection methods	Malicious nodes	Enhance the reliability of communication through the evaluation of the trustworthiness of vehicles and events.
[166]	2018			RL	Q-learning	Dishonest vehicle	Capable of resisting attacks and enhancing trust
[167]	2019			RL	Q-learning	Malicious node attacks	Minimize the vehicle’s privacy gain
[168]	2020			RL	Q-learning	Dishonest vehicle	Evaluate the trust value of the sender
[169]	2023			RL	DRL	OBU attacks	Reduce attack motives and make informed decisions to improve OBU collaboration.

## Data Availability

Not applicable.

## References

[1] Zantalis F., Koulouras G., Karabetsos S., Kandris D. (2019). A review of machine learning and IoT in smart transportation. Future Internet.

[2] Lee I., Lee K. (2015). The Internet of Things (IoT): Applications, investments, and challenges for enterprises. Bus. Horizons.

[3] Gubbi J., Buyya R., Marusic S., Palaniswami M. (2013). Internet of Things (IoT): A vision, architectural elements, and future directions. Future Gener. Comput. Syst..

[4] Bhardwaj I., Khara S. Research trends in architecture, security, services and applications of internet of vehicles (IOV). Proceedings of the 2018 International Conference on Computing, Power and Communication Technologies (GUCON).

[5] Gerla M., Lee E.K., Pau G., Lee U. Internet of vehicles: From intelligent grid to autonomous cars and vehicular clouds. Proceedings of the 2014 IEEE World Forum on Internet of Things (WF-IoT).

[6] Karagiannis G., Altintas O., Ekici E., Heijenk G., Jarupan B., Lin K., Weil T. (2011). Vehicular networking: A survey and tutorial on requirements, architectures, challenges, standards and solutions. IEEE Commun. Surv. Tutor..

[7] Ning H., Wang Z. (2011). Future internet of things architecture: Like mankind neural system or social organization framework?. IEEE Commun. Lett..

[8] Nitti M., Girau R., Floris A., Atzori L. On adding the social dimension to the internet of vehicles: Friendship and middleware. Proceedings of the 2014 IEEE International Black Sea Conference on Communications and Networking (BlackSeaCom).

[9] Alam K.M., Saini M., El Saddik A. (2015). Toward social internet of vehicles: Concept, architecture, and applications. IEEE Access.

[10] Cheng J., Cheng J., Zhou M., Liu F., Gao S., Liu C. (2015). Routing in internet of vehicles: A review. IEEE Trans. Intell. Transp. Syst..

[11] Datta S.K., Da Costa R.P.F., Härri J., Bonnet C. Integrating connected vehicles in Internet of Things ecosystems: Challenges and solutions. Proceedings of the 2016 IEEE 17th International Symposium on a World of Wireless, Mobile and Multimedia Networks (WoWMoM).

[12] Yang F., Li J., Lei T., Wang S. (2017). Architecture and key technologies for Internet of Vehicles: A survey. J. Commun. Inf. Networks.

[13] Li J.-L., Liu Z.-H., Yang F.-C. (2014). Internet of vehicles: The framework and key technology. J. Beijing Univ. Posts Telecommun..

[14] Yang F., Wang S., Li J., Liu Z., Sun Q. (2014). An overview of internet of vehicles. China Commun..

[15] Jiang C., Zhang H., Ren Y., Han Z., Chen K.C., Hanzo L. (2016). Machine learning paradigms for next-generation wireless networks. IEEE Wirel. Commun..

[16] Sun Y., Peng M., Zhou Y., Huang Y., Mao S. (2019). Application of machine learning in wireless networks: Key techniques and open issues. IEEE Commun. Surv. Tutor..

[17] Qayyum A., Usama M., Qadir J., Al-Fuqaha A. (2020). Securing connected & autonomous vehicles: Challenges posed by adversarial machine learning and the way forward. IEEE Commun. Surv. Tutor..

[18] Ghafari S.M., Beheshti A., Joshi A., Paris C., Mahmood A., Yakhchi S., Orgun M.A. (2020). A survey on trust prediction in online social networks. IEEE Access.

[19] Jøsang A., Ismail R., Boyd C. (2007). A survey of trust and reputation systems for online service provision. Decis. Support Syst..

[20] Håkansson P., Witmer H. (2015). Social media and trust: A systematic literature review. J. Bus. Econ..

[21] Truong N.B., Um T.W., Zhou B., Lee G.M. From personal experience to global reputation for trust evaluation in the social internet of things. Proceedings of the GLOBECOM 2017—2017 IEEE Global Communications Conference.

[22] Soleymani S.A., Abdullah A.H., Hassan W.H., Anisi M.H., Goudarzi S., Rezazadeh Baee M.A., Mandala S. (2015). Trust management in vehicular ad hoc network: A systematic review. EURASIP J. Wirel. Commun. Netw..

[23] Yu Y., Li K., Zhou W., Li P. (2012). Trust mechanisms in wireless sensor networks: Attack analysis and countermeasures. J. Netw. Comput. Appl..

[24] Mohammadi V., Rahmani A.M., Darwesh A.M., Sahafi A. (2019). Trust-based recommendation systems in Internet of Things: A systematic literature review. Hum. Centric Comput. Inf. Sci..

[25] Yan Z., Zhang P., Vasilakos A.V. (2016). A security and trust framework for virtualized networks and software-defined networking. Secur. Commun. Netw..

[26] Wang Y.-H. A trust management model for internet of vehicles. Proceedings of the 2020 4th International Conference on Cryptography, Security and Privacy.

[27] Jayasinghe U., Otebolaku A., Um T.W., Lee G.M. Data centric trust evaluation and prediction framework for IOT. Proceedings of the 2017 ITU Kaleidoscope: Challenges for a Data-Driven Society (ITU K).

[28] Mahmood A., Zhang W.E., Sheng Q.Z., Siddiqui S.A., Aljubairy A. (2019). Trust management for software-defined heterogeneous vehicular ad hoc networks. Security, Privacy and Trust in the IoT Environment.

[29] Mahmood A., Siddiqui S.A., Zhang W.E., Sheng Q.Z. A Hybrid Trust Management Model for Secure and Resource Efficient Vehicular Ad hoc Networks. Proceedings of the 2019 20th International Conference on Parallel and Distributed Computing, Applications and Technologies (PDCAT).

[30] Huang X., Yu R., Kang J., Zhang Y. (2017). Distributed reputation management for secure and efficient vehicular edge computing and networks. IEEE Access.

[31] Jayasinghe U., Lee G.M., Um T.W., Shi Q. (2018). Machine learning based trust computational model for IoT services. IEEE Trans. Sustain. Comput..

[32] Xia H., Xiao F., Zhang S.s., Hu C.q., Cheng X.z. Trustworthiness inference framework in the social Internet of Things: A context-aware approach. Proceedings of the IEEE INFOCOM 2019—IEEE Conference on Computer Communications.

[33] Lim J., Keum D., Ko Y.B. (2020). A stepwise and hybrid trust evaluation scheme for tactical wireless sensor networks. Sensors.

[34] Suo D., Sarma S.E. Real-time trust-building schemes for mitigating malicious behaviors in connected and automated vehicles. Proceedings of the 2019 IEEE Intelligent Transportation Systems Conference (ITSC).

[35] Al Falasi H., Mohamed N. (2015). Similarity-based trust management system for detecting fake safety messages in vanets. Proceedings of the Internet of Vehicles-Safe and Intelligent Mobility: Second International Conference, IOV 2015.

[36] Minhas U.F., Zhang J., Tran T., Cohen R. (2010). Towards expanded trust management for agents in vehicular ad-hoc networks. Int. J. Comput. Intell. Theory Pract..

[37] Li X., Liu J., Li X., Sun W. RGTE: A reputation-based global trust establishment in VANETs. Proceedings of the 2013 5th International Conference on Intelligent Networking and Collaborative Systems.

[38] Hu H., Lu R., Zhang Z., Shao J. (2016). REPLACE: A reliable trust-based platoon service recommendation scheme in VANET. IEEE Trans. Veh. Technol..

[39] Jordan M.I., Mitchell T.M. (2015). Machine learning: Trends, perspectives, and prospects. Science.

[40] Huang Y., Chen M. (2019). Improve reputation evaluation of crowdsourcing participants using multidimensional index and machine learning techniques. IEEE Access.

[41] Han G., He Y., Jiang J., Wang N., Guizani M., Ansere J.A. (2019). A synergetic trust model based on SVM in underwater acoustic sensor networks. IEEE Trans. Veh. Technol..

[42] Cherif A., Badhib A., Ammar H., Alshehri S., Kalkatawi M., Imine A. (2023). Credit card fraud detection in the era of disruptive technologies: A systematic review. J. King Saud Univ. Comput. Inf. Sci..

[43] Alsharif B., Altaher A.S., Altaher A., Ilyas M., Alalwany E. (2023). Deep Learning Technology to Recognize American Sign Language Alphabet. Sensors.

[44] Karimzadeh M., Vakanski A., Xian M., Zhang B. Post-Hoc Explainability of BI-RADS Descriptors in a Multi-Task Framework for Breast Cancer Detection and Segmentation. Proceedings of the 2023 IEEE 33rd International Workshop on Machine Learning for Signal Processing (MLSP).

[45] Rolnick D., Donti P.L., Kaack L.H., Kochanski K., Lacoste A., Sankaran K., Ross A.S., Milojevic-Dupont N., Jaques N., Waldman-Brown A. (2022). Tackling climate change with machine learning. ACM Comput. Surv. (CSUR).

[46] Ye H., Liang L., Li G.Y., Kim J., Lu L., Wu M. (2018). Machine learning for vehicular networks: Recent advances and application examples. IEEE Veh. Technol. Mag..

[47] Tan K., Bremner D., Le Kernec J., Zhang L., Imran M. (2022). Machine learning in vehicular networking: An overview. Digit. Commun. Netw..

[48] Tang Y., Cheng N., Wu W., Wang M., Dai Y., Shen X. (2019). Delay-minimization routing for heterogeneous VANETs with machine learning based mobility prediction. IEEE Trans. Veh. Technol..

[49] Li F., Song X., Chen H., Li X., Wang Y. (2018). Hierarchical routing for vehicular ad hoc networks via reinforcement learning. IEEE Trans. Veh. Technol..

[50] Yuan T., Da Rocha Neto W., Rothenberg C.E., Obraczka K., Barakat C., Turletti T. (2022). Machine learning for next-generation intelligent transportation systems: A survey. Trans. Emerg. Telecommun. Technol..

[51] Kim I.H., Bong J.H., Park J., Park S. (2017). Prediction of driver’s intention of lane change by augmenting sensor information using machine learning techniques. Sensors.

[52] Caruana R., Niculescu-Mizil A. An empirical comparison of supervised learning algorithms. Proceedings of the 23rd International Conference on Machine Learning.

[53] Alotaibi Y., Ilyas M. (2023). Ensemble-Learning Framework for Intrusion Detection to Enhance Internet of Things’ Devices Security. Sensors.

[54] Alloghani M., Al-Jumeily D., Mustafina J., Hussain A., Aljaaf A.J. (2020). A systematic review on supervised and unsupervised machine learning algorithms for data science. Supervised and Unsupervised Learning for Data Science.

[55] Akanksha E., Sharma N., Gulati K. Review on reinforcement learning, research evolution and scope of application. Proceedings of the 2021 5th International Conference on Computing Methodologies and Communication (ICCMC).

[56] Zhou Z., Oguz O.S., Leibold M., Buss M. (2020). A general framework to increase safety of learning algorithms for dynamical systems based on region of attraction estimation. IEEE Trans. Robot..

[57] Talpur A., Gurusamy M. (2021). Machine learning for security in vehicular networks: A comprehensive survey. IEEE Commun. Surv. Tutor..

[58] Sun Y., Wu L., Wu S., Li S., Zhang T., Zhang L., Xu J., Xiong Y. Security and Privacy in the Internet of Vehicles. Proceedings of the 2015 International Conference on Identification, Information, and Knowledge in the Internet of Things (IIKI).

[59] Bagga P., Das A.K., Wazid M., Rodrigues J.J., Park Y. (2020). Authentication protocols in internet of vehicles: Taxonomy, analysis, and challenges. IEEE Access.

[60] Samad A., Alam S., Mohammed S., Bhukhari M. Internet of vehicles (IoV) requirements, attacks and countermeasures. Proceedings of the 12th INDIACom—5th International Conference on Computing for Sustainable Global Development.

[61] Daeinabi A., Rahbar A.G. (2013). Detection of malicious vehicles (DMV) through monitoring in vehicular ad-hoc networks. Multimed. Tools Appl..

[62] Mokhtar B., Azab M. (2015). Survey on security issues in vehicular ad hoc networks. Alex. Eng. J..

[63] Sun Y., Wu L., Wu S., Li S., Zhang T., Zhang L., Xu J., Xiong Y., Cui X. (2017). Attacks and countermeasures in the internet of vehicles. Ann. Telecommun..

[64] Lu Z., Qu G., Liu Z. (2018). A survey on recent advances in vehicular network security, trust, and privacy. IEEE Trans. Intell. Transp. Syst..

[65] Sharma S., Kaushik B. (2019). A survey on internet of vehicles: Applications, security issues & solutions. Veh. Commun..

[66] Wang J., Jing X., Yan Z., Fu Y., Pedrycz W., Yang L.T. (2020). A survey on trust evaluation based on machine learning. ACM Comput. Surv. (CSUR).

[67] Garg T., Kagalwalla N., Churi P., Pawar A., Deshmukh S. (2020). A survey on security and privacy issues in IoV. Int. J. Electr. Comput. Eng. (2088-8708).

[68] Rehman A., Hassan M.F., Yew K.H., Paputungan I., Tran D.C. (2020). State-of-the-art IoV trust management a meta-synthesis systematic literature review (SLR). PeerJ Comput. Sci..

[69] Sharma S., Kaushik B. (2021). A survey on nature-inspired algorithms and its applications in the Internet of Vehicles. Int. J. Commun. Syst..

[70] Mchergui A., Moulahi T., Zeadally S. (2022). Survey on artificial intelligence (AI) techniques for vehicular ad-hoc networks (VANETs). Veh. Commun..

[71] Guo J., Bilal M., Qiu Y., Qian C., Xu X., Choo K.K.R. (2022). Survey on digital twins for Internet of Vehicles: Fundamentals, challenges, and opportunities. Digit. Commun. Netw..

[72] Abuarqoub A., Alzu’bi A., Hammoudeh M., Ahmad A., Al-Shargabi B. A Survey on Vehicular Ad hoc Networks Security Attacks and Countermeasures. Proceedings of the 6th International Conference on Future Networks & Distributed Systems.

[73] Garg A., Chauhan A., Shambharkar P.G. Security Threats & Attacks in IoV Environment: Open Research Issues and Challenges. Proceedings of the 2022 Third International Conference on Intelligent Computing Instrumentation and Control Technologies (ICICICT).

[74] Hasrouny H., Samhat A.E., Bassil C., Laouiti A. (2017). VANet security challenges and solutions: A survey. Veh. Commun..

[75] Fadhil J.A., Sarhan Q.I. Internet of Vehicles (IoV): A survey of challenges and solutions. Proceedings of the 2020 21st International Arab Conference on Information Technology (ACIT).

[76] Uprety A., Rawat D.B. (2020). Reinforcement learning for iot security: A comprehensive survey. IEEE Internet Things J..

[77] Tanwar S., Vora J., Tyagi S., Kumar N., Obaidat M.S. (2018). A systematic review on security issues in vehicular ad hoc network. Secur. Priv..

[78] Shahid M.A., Jaekel A., Ezeife C., Al-Ajmi Q., Saini I. Review of potential security attacks in VANET. Proceedings of the 2018 Majan International Conference (MIC).

[79] Cheng X., Zhang R., Yang L. (2018). Wireless toward the era of intelligent vehicles. IEEE Internet Things J..

[80] Zhang W., Xi X. (2016). The innovation and development of Internet of Vehicles. China Commun..

[81] Zheng K., Zheng Q., Chatzimisios P., Xiang W., Zhou Y. (2015). Heterogeneous vehicular networking: A survey on architecture, challenges, and solutions. IEEE Commun. Surv. Tutor..

[82] Contreras-Castillo J., Zeadally S., Guerrero Ibáñez J.A. (2017). A seven-layered model architecture for Internet of Vehicles. J. Inf. Telecommun..

[83] Darwish T.S., Bakar K.A. (2018). Fog based intelligent transportation big data analytics in the internet of vehicles environment: Motivations, architecture, challenges, and critical issues. IEEE Access.

[84] Lopez H.J.D., Siller M., Huerta I. Internet of vehicles: Cloud and fog computing approaches. Proceedings of the 2017 IEEE International Conference on Service Operations and Logistics, and Informatics (SOLI).

[85] Liu N. (2011). Internet of Vehicles: Your next connection. Huawei WinWin.

[86] Gandotra P., Jha R.K., Jain S. (2017). A survey on device-to-device (D2D) communication: Architecture and security issues. J. Netw. Comput. Appl..

[87] Liu K., Xu X., Chen M., Liu B., Wu L., Lee V.C. (2019). A hierarchical architecture for the future internet of vehicles. IEEE Commun. Mag..

[88] Wu W., Yang Z., Li K. (2016). Internet of Vehicles and applications. Internet of Things.

[89] Wan J., Zhang D., Zhao S., Yang L.T., Lloret J. (2014). Context-aware vehicular cyber-physical systems with cloud support: Architecture, challenges, and solutions. IEEE Commun. Mag..

[90] Mahmood Z. (2020). Connected vehicles in the IoV: Concepts, technologies and architectures. Connected Vehicles in the Internet of Things: Concepts, Technologies and Frameworks for the IoV.

[91] Peng H. (2016). Connected and automated vehicles: The roles of dynamics and control. Mech. Eng..

[92] Qureshi K.N., Din S., Jeon G., Piccialli F. (2020). Internet of vehicles: Key technologies, network model, solutions and challenges with future aspects. IEEE Trans. Intell. Transp. Syst..

[93] Thakur A., Malekian R. (2019). Fog computing for detecting vehicular congestion, an internet of vehicles based approach: A review. IEEE Intell. Transp. Syst. Mag..

[94] Huang C., Lu R., Choo K.K.R. (2017). Vehicular fog computing: Architecture, use case, and security and forensic challenges. IEEE Commun. Mag..

[95] Wazid M., Bagga P., Das A.K., Shetty S., Rodrigues J.J., Park Y. (2019). AKM-IoV: Authenticated key management protocol in fog computing-based Internet of vehicles deployment. IEEE Internet Things J..

[96] Vishwanath A., Peruri R., He J.S. (2016). Security in Fog Computing through Encryption.

[97] Khan S., Parkinson S., Qin Y. (2017). Fog computing security: A review of current applications and security solutions. J. Cloud Comput..

[98] Lin C.C., Deng D.J., Yao C.C. (2017). Resource allocation in vehicular cloud computing systems with heterogeneous vehicles and roadside units. IEEE Internet Things J..

[99] Xu W., Shi W., Lyu F., Zhou H., Cheng N., Shen X. (2019). Throughput analysis of vehicular internet access via roadside WiFi hotspot. IEEE Trans. Veh. Technol..

[100] Gür G., Bahtiyar Ş., Alagöz F. (2015). Security analysis of computer networks: Key concepts and methodologies. Modeling and Simulation of Computer Networks and Systems.

[101] Kim S. (2018). Blockchain for a trust network among intelligent vehicles. Advances in Computers.

[102] Tangade S.S., Manvi S.S. A survey on attacks, security and trust management solutions in VANETs. Proceedings of the 2013 Fourth International Conference on Computing, Communications and Networking Technologies (ICCCNT).

[103] Manvi S.S., Tangade S. (2017). A survey on authentication schemes in VANETs for secured communication. Veh. Commun..

[104] Sharma N., Chauhan N., Chand N. Security challenges in Internet of Vehicles (IoV) environment. Proceedings of the 2018 First International Conference on Secure Cyber Computing and Communication (ICSCCC).

[105] La V.H., Cavalli A.R. (2014). Security attacks and solutions in vehicular ad hoc networks: A survey. Int. J. AdHoc Netw. Syst. (IJANS).

[106] Rawat A., Sharma S., Sushil R. (2012). VANET: Security attacks and its possible solutions. J. Inf. Oper. Manag..

[107] Kim Y., Kim I., Shim C.Y. A taxonomy for DOS attacks in VANET. Proceedings of the 2014 14th International Symposium on Communications and Information Technologies (ISCIT).

[108] Bariah L., Shehada D., Salahat E., Yeun C.Y. Recent advances in VANET security: A survey. Proceedings of the 2015 IEEE 82nd Vehicular Technology Conference (VTC2015-Fall).

[109] Gai F., Zhang J., Zhu P., Jiang X. (2017). Ratee-based trust management system for internet of vehicles. Proceedings of the Wireless Algorithms, Systems, and Applications: 12th International Conference, WASA 2017.

[110] Ahmad F., Kurugollu F., Adnane A., Hussain R., Hussain F. (2020). MARINE: Man-in-the-middle attack resistant trust model in connected vehicles. IEEE Internet Things J..

[111] Arellano W., Mahgoub I. TrafficModeler extensions: A case for rapid VANET simulation using, OMNET++, SUMO, and VEINS. Proceedings of the 2013 High Capacity Optical Networks and Emerging/Enabling Technologies.

[112] Krajzewicz D., Erdmann J., Behrisch M., Bieker L. (2012). Recent development and applications of SUMO-Simulation of Urban MObility. Int. J. Adv. Syst. Meas..

[113] Sommer C., Eckhoff D., Brummer A., Buse D.S., Hagenauer F., Joerer S., Segata M. (2019). Veins: The open source vehicular network simulation framework. Recent Advances in Network Simulation: The OMNeT++ Environment and Its Ecosystem.

[114] Zhang J., Zheng K., Zhang D., Yan B. (2020). AATMS: An anti-attack trust management scheme in VANET. IEEE Access.

[115] Guleng S., Wu C., Chen X., Wang X., Yoshinaga T., Ji Y. (2019). Decentralized trust evaluation in vehicular Internet of Things. IEEE Access.

[116] Rehmani M.H., Saleem Y. (2015). Network simulator NS-2. Encyclopedia of Information Science and Technology.

[117] Joy J., Rabsatt V., Gerla M. (2018). Internet of Vehicles: Enabling safe, secure, and private vehicular crowdsourcing. Internet Technol. Lett..

[118] Yao Y., Xiao B., Wu G., Liu X., Yu Z., Zhang K., Zhou X. (2018). Multi-channel based Sybil attack detection in vehicular ad hoc networks using RSSI. IEEE Trans. Mob. Comput..

[119] Tayeb S., Pirouz M., Esguerra G., Ghobadi K., Huang J., Hill R., Lawson D., Li S., Zhan T., Zhan J. Securing the positioning signals of autonomous vehicles. Proceedings of the 2017 IEEE International Conference on Big Data (Big Data).

[120] Abbas S., Faisal M., Rahman H.U., Khan M.Z., Merabti M. (2018). Masquerading attacks detection in mobile ad hoc networks. IEEE Access.

[121] Rawat D.B., Garuba M., Chen L., Yang Q. (2017). On the security of information dissemination in the Internet-of-Vehicles. Tsinghua Sci. Technol..

[122] Wang C., Zhao Z., Gong L., Zhu L., Liu Z., Cheng X. (2018). A distributed anomaly detection system for in-vehicle network using HTM. IEEE Access.

[123] Dua A., Kumar N., Das A.K., Susilo W. (2017). Secure message communication protocol among vehicles in smart city. IEEE Trans. Veh. Technol..

[124] Osibo B.K., Zhang C., Xia C., Zhao G., Jin Z. (2021). Security and privacy in 5G internet of vehicles (IoV) environment. J. Internet Things.

[125] Kumar S., Mann K.S. Prevention of DoS attacks by detection of multiple malicious nodes in VANETs. Proceedings of the 2019 International Conference on Automation, Computational and Technology Management (ICACTM).

[126] Ahmad F., Adnane A., Franqueira V.N., Kurugollu F., Liu L. (2018). Man-in-the-middle attacks in vehicular ad-hoc networks: Evaluating the impact of attackers’ strategies. Sensors.

[127] Huang X., Yu R., Pan M., Shu L. (2018). Secure roadside unit hotspot against eavesdropping based traffic analysis in edge computing based internet of vehicles. IEEE Access.

[128] Baruah B., Dhal S. A Secure and privacy-preserved road condition monitoring system. Proceedings of the 2020 International Conference on COMmunication Systems & NETworkS (COMSNETS).

[129] Vadhana Kumari S., Paramasivan B. (2017). Defense against Sybil attacks and authentication for anonymous location-based routing in MANET. Wirel. Netw..

[130] Andrea I., Chrysostomou C., Hadjichristofi G. Internet of Things: Security vulnerabilities and challenges. Proceedings of the 2015 IEEE Symposium on Computers and Communication (ISCC).

[131] Feltus C. (2020). Current and future RL’s contribution to emerging network security. Procedia Comput. Sci..

[132] Biswas S.K. (2018). Intrusion detection using machine learning: A comparison study. Int. J. Pure Appl. Math..

[133] Wu M., Song Z., Moon Y.B. (2019). Detecting cyber-physical attacks in CyberManufacturing systems with machine learning methods. J. Intell. Manuf..

[134] Nguyen T.T., Reddi V.J. (2021). Deep reinforcement learning for cyber security. IEEE Trans. Neural Netw. Learn. Syst..

[135] Gyawali S., Qian Y. Misbehavior detection using machine learning in vehicular communication networks. Proceedings of the ICC 2019–2019 IEEE International Conference on Communications (ICC).

[136] Singh P.K., Gupta R.R., Nandi S.K., Nandi S. (2019). Machine learning based approach to detect wormhole attack in VANETs. Web, Artificial Intelligence and Network Applications: Proceedings of the Workshops of the 33rd International Conference on Advanced Information Networking and Applications (WAINA-2019), Matsue, Japan, 27–29 March 2019.

[137] Kumar S., Singh K., Kumar S., Kaiwartya O., Cao Y., Zhou H. (2019). Delimitated anti jammer scheme for Internet of vehicle: Machine learning based security approach. IEEE Access.

[138] Sharma P., Liu H. (2020). A machine-learning-based data-centric misbehavior detection model for internet of vehicles. IEEE Internet Things J..

[139] Sharma A., Babbar H., Sharma A. Ton-iot: Detection of attacks on internet of things in vehicular networks. Proceedings of the 2022 6th International Conference on Electronics, Communication and Aerospace Technology.

[140] Alalwany E., Mahgoub I. (2022). Classification of Normal and Malicious Traffic Based on an Ensemble of Machine Learning for a Vehicle CAN-Network. Sensors.

[141] Anyanwu G.O., Nwakanma C.I., Lee J.M., Kim D.S. (2023). Falsification Detection System for IoV Using Randomized Search Optimization Ensemble Algorithm. IEEE Trans. Intell. Transp. Syst..

[142] Lokman S.F., Othman A.T., Musa S., Abu Bakar M.H. (2019). Deep contractive autoencoder-based anomaly detection for in-vehicle controller area network (CAN). Progress in Engineering Technology: Automotive, Energy Generation, Quality Control and Efficiency.

[143] D’Angelo G., Castiglione A., Palmieri F. (2020). A cluster-based multidimensional approach for detecting attacks on connected vehicles. IEEE Internet Things J..

[144] El Attar A., Fadlallah A., Chbib F., Khatoun R. Unsupervised Learning Algorithms for Denial of Service Detection in Vehicular Networks. Proceedings of the 2022 International Conference on Electrical, Computer, Communications and Mechatronics Engineering (ICECCME).

[145] Valentini E.P., Rocha Filho G.P., De Grande R.E., Ranieri C.M., Pereira L.A., Meneguette R.I. (2023). A Novel Mechanism for Misbehaviour Detection in Vehicular Networks. IEEE Access.

[146] Lu X., Wan X., Xiao L., Tang Y., Zhuang W. Learning-based rogue edge detection in VANETs with ambient radio signals. Proceedings of the 2018 IEEE International Conference on Communications (ICC).

[147] Gu T., Abhishek A., Fu H., Zhang H., Basu D., Mohapatra P. Towards learning-automation IoT attack detection through reinforcement learning. Proceedings of the 2020 IEEE 21st International Symposium on “A World of Wireless, Mobile and Multimedia Networks” (WoWMoM).

[148] Chinchali S., Hu P., Chu T., Sharma M., Bansal M., Misra R., Pavone M., Katti S. Cellular network traffic scheduling with deep reinforcement learning. Proceedings of the AAAI Conference on Artificial Intelligence.

[149] Ferdowsi A., Saad W. (2018). Deep learning for signal authentication and security in massive internet-of-things systems. IEEE Trans. Commun..

[150] Lopez-Martin M., Carro B., Sanchez-Esguevillas A. (2020). Application of deep reinforcement learning to intrusion detection for supervised problems. Expert Syst. Appl..

[151] Sherazi H.H.R., Iqbal R., Ahmad F., Khan Z.A., Chaudary M.H. (2019). DDoS attack detection: A key enabler for sustainable communication in internet of vehicles. Sustain. Comput. Informatics Syst..

[152] Xu Y., Lei M., Li M., Zhao M., Hu B. A new anti-jamming strategy based on deep reinforcement learning for MANET. Proceedings of the 2019 IEEE 89th Vehicular Technology Conference (VTC2019-Spring).

[153] Sedar R., Kalalas C., Vázquez-Gallego F., Alonso-Zarate J. Reinforcement learning based misbehavior detection in vehicular networks. Proceedings of the ICC 2022—IEEE International Conference on Communications.

[154] Talpur A., Gurusamy M. (2023). On Attack-Resilient Service Placement and Availability in Edge-Enabled IoV Networks. IEEE Trans. Intell. Transp. Syst..

[155] Eziama E., Tepe K., Balador A., Nwizege K.S., Jaimes L.M. Malicious node detection in vehicular ad-hoc network using machine learning and deep learning. Proceedings of the 2018 IEEE Globecom Workshops (GC Wkshps).

[156] Eziama E., Ahmed S., Ahmed S., Awin F., Tepe K. Detection of adversary nodes in machine-to-machine communication using machine learning based trust model. Proceedings of the 2019 IEEE International Symposium on Signal Processing and Information Technology (ISSPIT).

[157] Montenegro J., Iza C., Aguilar Igartua M. Detection of position falsification attacks in VANETs applying trust model and machine learning. Proceedings of the 17th ACM Symposium on Performance Evaluation of Wireless Ad Hoc Sensor, & Ubiquitous Networks.

[158] Ghaleb A.F., Saeed F., Al-Sarem M., Ali Saleh Al-rimy B., Boulila W., Eljialy A.E.M., Aloufi K., Alazab M. (2020). Misbehavior-aware on-demand collaborative intrusion detection system using distributed ensemble learning for VANET. Electronics.

[159] Manogaran G., Rawal B.S. Machine learning based trust model for secure internet of vehicle data exchange. Proceedings of the 2020 IEEE Globecom Workshops (GC Wkshps).

[160] Faisal S.M., Gupta B.K., Zaidi T. A hybrid framework to prevent VANET from Sybil Attack. Proceedings of the 2022 5th International Conference on Multimedia, Signal Processing and Communication Technologies (IMPACT).

[161] Siddiqui S.A., Mahmood A., Sheng Q.Z., Suzuki H., Ni W. (2023). Towards a Machine Learning Driven Trust Management Heuristic for the Internet of Vehicles. Sensors.

[162] Kamel J., Haidar F., Jemaa I.B., Kaiser A., Lonc B., Urien P. A misbehavior authority system for sybil attack detection in c-its. Proceedings of the 2019 IEEE 10th Annual Ubiquitous Computing, Electronics & Mobile Communication Conference (UEMCON).

[163] Tangade S., Manvi S.S., Hassan S. A deep learning based driver classification and trust computation in VANETs. Proceedings of the 2019 IEEE 90th Vehicular Technology Conference (VTC2019-Fall).

[164] Siddiqui S.A., Mahmood A., Zhang W.E., Sheng Q.Z. (2019). Machine learning based trust model for misbehaviour detection in internet-of-vehicles. Neural Information Processing: 26th International Conference, ICONIP 2019, Sydney, NSW, Australia, 12–15 December 2019.

[165] Huang F., Li Q., Zhao J. Trust Management Model of VANETs Based on Machine Learning and Active Detection Technology. Proceedings of the 2022 IEEE/CIC International Conference on Communications in China (ICCC Workshops).

[166] Aref A., Tran T. (2018). A hybrid trust model using reinforcement learning and fuzzy logic. Comput. Intell..

[167] Wang W., Min M., Xiao L., Chen Y., Dai H. Protecting semantic trajectory privacy for VANET with reinforcement learning. Proceedings of the ICC 2019–2019 IEEE International Conference on Communications (ICC).

[168] Guo J., Li X., Liu Z., Ma J., Yang C., Zhang J., Wu D. (2020). TROVE: A context-awareness trust model for VANETs using reinforcement learning. IEEE Internet Things J..

[169] Zhang B., Wang X., Xie R., Li C., Zhang H., Jiang F. (2023). A reputation mechanism based Deep Reinforcement Learning and blockchain to suppress selfish node attack motivation in Vehicular Ad-Hoc Network. Future Gener. Comput. Syst..

[170] Kachalsky I., Zakirzyanov I., Ulyantsev V. Applying reinforcement learning and supervised learning techniques to play hearthstone. Proceedings of the 2017 16th IEEE International Conference on Machine Learning and Applications (ICMLA).

[171] García J., Majadas R., Fernández F. (2020). Learning adversarial attack policies through multi-objective reinforcement learning. Eng. Appl. Artif. Intell..

[172] Qu X., Sun Z., Ong Y.S., Gupta A., Wei P. (2020). Minimalistic attacks: How little it takes to fool deep reinforcement learning policies. IEEE Trans. Cogn. Dev. Syst..

